# Positive and Relaxed Selective Pressures Have Both Strongly Influenced the Evolution of Cryonotothenioid Fishes during Their Radiation in the Freezing Southern Ocean

**DOI:** 10.1093/gbe/evad049

**Published:** 2023-03-23

**Authors:** Kevin T Bilyk, Xuan Zhuang, Chiara Papetti

**Affiliations:** Department of Biology, Montclair State University, New Jersey; Department of Biological Sciences, University of Arkansas, Fayetteville; Department of Biology, University of Padova, Italy

**Keywords:** cold adaptation, cold specialization, molecular evolution, notothenioids

## Abstract

Evolution in the chronic cold of the Southern Ocean has had a profound influence on the physiology of cryonotothenioid fishes. However, the suite of genetic changes underlying the physiological gains and losses in these fishes is still poorly surveyed. By identifying the genomic signatures of selection, this study aims to identify the functional classes of genes that have been changed following two major physiological transitions: the onset of freezing temperatures and the loss of hemoproteins. Looking at the changes that followed the onset of freezing temperatures, positive selective pressure was found among a set of broadly acting gene regulatory factors, suggesting a route through which cryonotothenioid gene expression has been retooled for life in the cold. Further, genes related to the cell cycle and cellular adhesion were found under positive selection suggesting that both present key challenges to life in freezing waters. By contrast, genes showing signatures of the relaxation of selective pressure showed a narrower biological impact, acting on genes related to mitochondrial function. Finally, although chronic cold-water temperatures appear correlated with substantial genetic change, the loss of hemoproteins resulted in little observable change in protein-coding genes relative to their red-blooded relatives. Combined, the influence of positive and relaxed selection shows that long-term exposure to cold has led to profound changes in cryonotothenioid genomes that may make it challenging for them to adapt to a rapidly changing climate.

**Significance**

Evolution in the isolation of the cold Southern Ocean has led to widespread physiological change in the cryonotothenioid fishes, but how this is reflected in changes among their pool of protein-coding genes remains poorly described. Here, we comprehensively investigated changes in the suite of protein-coding genes to identify which genes and associated functional categories have been changed following evolution in the chronic cold.

## Introduction

Today, the waters of the Antarctic shelf are dominated by the members of a single taxonomic group, the cryonotothenioid fishes, comprised of the five Antarctic families of the perciform suborder Notothenioidei (Eastman 2005; Dornburg et al. 2017; Near et al. 2018). The origin of this group is closely linked to the onset of freezing conditions in Antarctic waters (Matschiner et al. 2011) and their ubsequent radiation driven by continued changes to the region's climate (Near et al. 2012). Evolution in this isolated, frigid environment has resulted in a diverse group of fishes that now share a profound specialization to life in the cold (Beers and Jayasundara 2015; Daane and Detrich 2022). However, the genetic consequences of evolution in chronic cold remain poorly surveyed in this group.

The most obvious shift in selective pressure experienced by the cryonotothenioids during the geological evolution of the Southern Ocean came from the region's cooling. In contrast to the cold-temperate waters inhabited by their closest non-Antarctic relatives, surface water temperatures around Antarctica remain below −1.5 °C, and freezing water temperatures define the most species-rich waters along the Antarctic coast, even to great depths (DeVries and Steffensen 2005). In addition to being cold, the waters around Antarctica are remarkable for stability, with only modest thermal variability compared with temperate and tropical waters (Barnes et al. 2006). This reaches an extreme in high-latitude habitats, such as the waters of McMurdo Sound, which are characterized by water temperatures that remain near their freezing point throughout the year (Cziko et al. 2014).

Although the origin of the cryonotothenioid clade is estimated at 22.4 million years ago (Ma) (Near et al. 2012), most of the living diversity of Antarctic notothenioids is believed to have originated more recently, with many speciation events taking place within the last 5 Ma (Bista et al. 2022). We expect that evolution in these freezing waters would have imposed strong selective pressures to deal with the biological challenges and opportunities that came with life in the cold, but it would also have exposed endemic fishes to an important relaxation of selective pressure for some biological traits less impacted by cold adaptation. Specifically, the persistence of cold-stable water temperatures and high dissolved oxygen levels would be expected to relax selective pressures across biological systems that previously dealt with temperature and oxygen variability (Somero 2010).

In addition to the influence of temperature, the isolation of the Southern Ocean has allowed the evolution of lineages characterized by substantial and, in some cases unique, physiological reorganization. This is exemplified by the members of the notothenioid family Channichthyidae (the icefishes) that are extraordinary for the absence of the respiratory pigment hemoglobin (Sidell and O'Brien 2006). Oxygen is found solely in physical solution in icefish blood, and these fishes are thought capable of surviving in the Southern Ocean because the cold, stable, and well-mixed Antarctic waters create a marine environment rich with dissolved oxygen. Although icefishes have undergone widespread and profound anatomical and physiological reorganization following the loss of hemoglobin, whether this is mirrored in large changes to the protein pool and corresponding genes, or if it is compensated through plasticity and gene expression regulation (Bargelloni et al. 2019), is less understood. At a minimum, the loss of hemoglobin would be expected to produce a relaxation of purifying selection on its former partnering genes, and prior work has suggested that at least some genes with roles focused solely on supporting hemoglobin may be heading towards becoming pseudogenes (Bilyk et al. 2019).

Several prior studies have aimed to develop a broad understanding of the changes to protein-coding genes in the cryonotothenioids associated with polar evolution. Looking first at global biases in amino-acid composition that may explain adaptation to polar conditions, Berthelot et al. (2019) found evidence for only limited change, identifying an increase in leucine substitutions for methionine. This change was hypothesized to serve as an adaptation in the cryonotothenioids tied to redox regulation, providing a defense against increased levels of reactive oxygen species in the oxygen-rich waters of the Southern Ocean.

Studies by Daane et al. (2019, 2020) have since investigated how genetic mechanisms have shaped evolutionary change in the cryonotothenioids during their radiation in the Southern Ocean. Using the evolution of buoyancy adaptation among several Antarctic lineages (Eastman 2020) as a model to explore the origins of novel traits in the cryonotothenioids, this was found to result from bone demineralization associated with developmental alterations similar to human skeletal dysplasia. However, the onset of positive diversifying selective pressure that reshaped these pathways was found to precede the origin of the cryonotothenioids, showing the role of historical contingency in shaping the capacity for adaptation in this group (Daane et al. 2019). Contrasting with buoyancy adaptation, Daane et al. (2020) investigated the genetic regions controlling erythropoiesis and found evidence that relaxation of selective pressure followed sustained cooling of the Southern Ocean and acted independently on these genetic elements among several impacted lineages of cryonotothenioids. The influence of this relaxation of selective pressure was further found biased toward conserved noncoding elements (CNEs) rather than to coding regions.

Although results by Berthelot et al. (2019) and Daane et al. (2019, 2020) provide important insight into the timing and mechanisms of adaptive change in the cryonotothenioids, how the suite of protein-coding genes have been changed during their radiation in the freezing Southern Ocean remains poorly surveyed. The aim of this study is therefore to identify how changes in selective pressure following the onset of chronic cold conditions and following the loss of hemoproteins have shaped the suite of protein-coding genes of modern cryonotothenioid fishes.

## Results

**Fig. 1.**
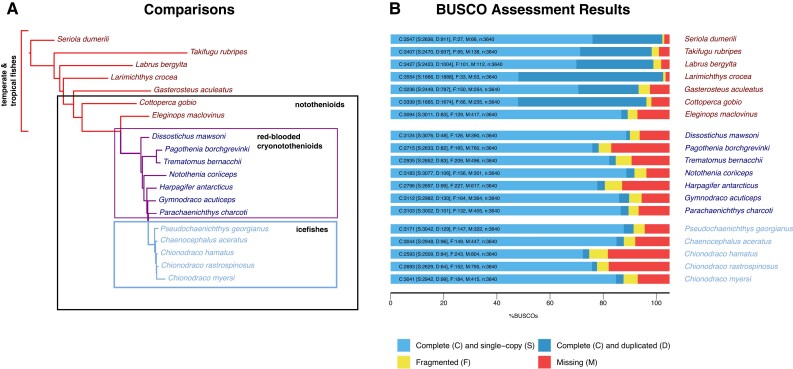
Panel (*A*) shows the comparative groupings used in the analysis, with species in red highlighting temperate/tropical fishes, species in purple highlighting red-blooded cryonotothenioids, and species in blue highlighting icefishes. Panel (*B*) reports BUSCO metrics for the peptide sets used for original ortholog detection in all 19 target species.

To investigate how the shifts in selective pressure from evolution in chronic cold and loss of hemoproteins have affected the pool of protein-coding genes among the cryonotothenioids, we obtained and analyzed 3,453 orthogroups that contained all species in a 19-taxa data set (fig. 1). These taxa included seven red-blooded cryonotothenioids and five hemoglobin-lacking icefishes where public genomic and transcriptomic resources were available, along with a background set of seven temperate and tropical fishes. The 3,453 orthogroups that were used in the analysis represent 15.3% of the predicted peptides identified in the annotated *Dissostichus mawsoni* genome. Metrics on the filtered genomic and transcriptomic resources used to identify orthogroups are presented in figure 1, alongside the phylogenetic framework for evolutionary hypothesis testing (detailed in supplementary fig. S1, Supplementary Material online). Additional information on the tissue content of each transcriptome is presented in supplementary figure S2, Supplementary Material online, and comparisons between the genomic- and transcriptomic-derived predicted peptides for species, where both were available, are presented in supplementary figure S3, Supplementary Material online.

This set of orthogroups was used for alignment construction then evaluated using HyPhy to identify genes showing signatures of either positive or relaxed selective pressures. The goal being to understand whether positive and relaxed selective pressures affected distinct functional categories of protein-coding genes.

**Fig. 2.**
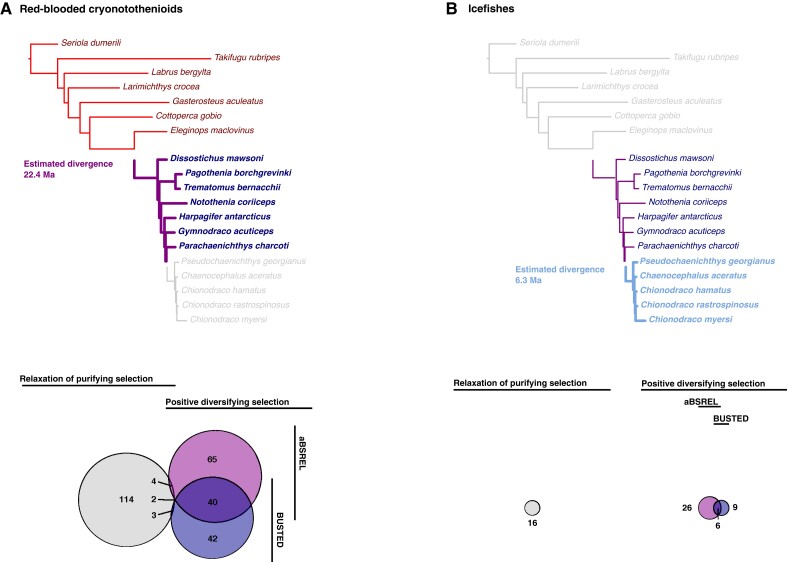
Number and distribution of orthogroups showing changes in selective pressure with the transition to freezing water temperatures (A) and with hemoprotein loss (B). The phylogenetic trees show the comparison each analysis is based on while the Venn diagrams show the number of genes identified by RELAX as being under relaxation of purifying selective pressure, or by aBSREL and BUSTED as being under positive selective pressure. Estimated divergence times for the two focal clades are from Near et al. (2012).

First, the red-blooded cryonotothenioids were compared with temperate and tropical fishes to identify the suite of changes that have followed their evolution in chronic cold. From this comparison, adaptive Branch-Site Random Effects Likelihood (aBSREL) and Branch-Site Unrestricted Statistical Test for Episodic Diversification (BUSTED) identified 113 and 89 orthogroups respectively under positive selection, with 160 orthogroups identified as experiencing positive selective pressure from either of the two analyses, and 42 orthogroups consistently identified as experiencing positive selective pressure by both analyses (fig. 2*A*). RELAX then identified 114 orthogroups as experiencing relaxed selective pressure (fig. 2*A*). The set of genes showing signatures of positive selective pressure and relaxed purifying selective pressure was largely distinct, but nine genes were found to show signatures of both (supplementary table S3, Supplementary Material online).

Next, the icefishes were compared against a background set of the red-blooded cryonotothenioids, to identify genes under changed selective pressure following the loss of hemoproteins. This comparison showed a far smaller signature of impacts from both positive and relaxed selective pressures. The signature of significant positive selective pressure was identified on only 15 orthogroups using BUSTED, and 32 orthogroups with aBSREL, resulting in a total of 41 distinct orthogroups identified as experiencing positive selective pressure from either analysis, with six orthogroups identified under positive selective pressure by both analyses (fig. 2*B*). Similarly, relaxed selective pressure was detected for only 16 orthogroups using RELAX (fig. 2*B*). There was no overlap among these sets of genes showing signatures of positive and relaxed selective pressures.

**Fig. 3.**
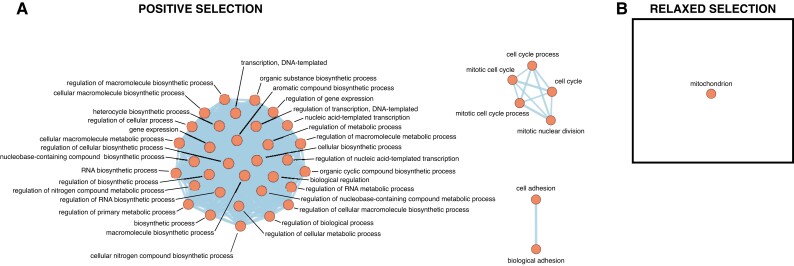
GO terms enriched in the BP ontology among the red-blooded cryonotothenioids among genes showing signatures of positive (*A*) and relaxed (*B*) selective pressures.

To put changes in selective pressure into their broader biological context, we tested for functional enrichment of genes showing signatures of changed selective pressure by Gene Ontology (GO) enrichment analysis (supplementary tables S4–9, Supplementary Material online). Enrichment analysis of the combined set of 160 orthogroups that were identified under positive selection in the red-blooded cryonotothenioids compared with temperate and tropical fishes by either aBSREL (supplementary table S4, Supplementary Material online) or BUSTED (supplementary table S5, Supplementary Material online) found a total of 40 significant enriched GO terms at the false discovery rate (FDR)-corrected *P*-value threshold of 0.1 (supplementary table S6, Supplementary Material online). Clustering through EnrichmentMap identified three clusters of GO terms connected by shared sets of genes (fig. 3*A*), identifying functional impacts on the regulation of gene expression, cell cycle progression, and cell adhesion. Looking at the more restricted set of 42 orthogroups where both BUSTED and aBSREL agreed they were under positive selection found 31 significantly enriched GO terms (supplementary table S7, Supplementary Material online). Although this shared set of orthogroups identified by both BUSTED and aBSREL provides a narrower view of changed biological systems in the cryonotothenioids, it continues to highlight a critical role of changed regulation of gene expression. This set of 42 orthogroups identified as experiencing positive selective pressure by both BUSTED and aBSREL is provided in supplementary table S8, Supplementary Material online, while the larger set of 160 orthogroups identified by either of those analyses is provided in supplementary table S9, Supplementary Material online.

In contrast with the investigation of orthogroups showing signatures of positive selective pressure, enrichment analysis of the orthogroups under relaxed selective pressure in the red-blooded cryonotothenioids produced a much more restricted set of enriched GO terms after FDR correction (supplementary table S10, Supplementary Material online). Only a single GO term was enriched, identifying the mitochondria as a key site of relaxed selective pressure. The full set of orthogroups identified by RELAX as experiencing significant relaxation of selective pressure is presented in supplementary table S11, Supplementary Material online. Further, the set of genes found under positive or relaxed selection is grouped by cluster and GO term in supplementary tables S12–15, Supplementary Material online.

Unlike the comparison between the red-blooded cryonotothenioids and temperate fishes, only a small number of orthogroups were identified under changed selective pressure in the icefishes relative to the red-blooded cryonotothenioids. At the target FDR-corrected *P* value, these did not result in any enriched GO terms. Instead, the biological impact of changed protein-coding sequences among the icefishes was investigated by direct interrogation of the set of orthogroups, and to help characterize their disparate biological roles, they were grouped according to their Panther protein classification (Thomas et al. 2003) as presented in table 1.

**Table 1:** Genes Identified under Changed Selection in the Icefishes (Family Channichthyidae)

Panther Protein Classification	aBSREL	BUSTED	RELAX (*K* < 1)
**Aminoacyl-tRNA synthetase**			*LARS2*
**Apolipoprotein**	***APOA1***	***APOA1***	
**ATP-binding cassette (ABC) transporter**	*ABCB8*		
**ATP synthase**			*ATP6V1F*
**Carbohydrate phosphatase**		*FBP1*	
**Chromatin/chromatin-binding protein**		*NIPBL*	
**Cysteine protease**	*CAPN1*		
**Dehydrogenase**			*DHRS12*
**Extracellular matrix structural protein**	*SNRNP48*		
**G-protein–coupled receptor**	*LPAR4*		
**Gap junction**	*CX30.3*		
**Glycosyltransferase**	*B3GNT3*		
**Heterotrimeric G-protein**	*GNAO1B*, ***GNB1B***	***GNB1B***	
**Histone modifying enzyme**	***SIRT5***	***SIRT5***	
**Homeodomain transcription factor**	*IRX3A*		
**Ion channel**			*KCNK5A*
**Ligand-gated ion channel**	*GABRR1*		
**MADS box transcription factor**	*MEF2CB*		
**Membrane traffic protein**	*MX1*		
**Metalloprotease**		*ACE*	
**mRNA polyadenylation factor**			*TOE1*
**Nonreceptor serine/threonine protein kinase**	*MAK*, *NEK3*		
**Primary active transporter**			*DERlL*
**Protein modifying enzyme**	***RPS6KB1***	*PRMT8*, ***RPS6KB1***	*JMJD7*, *ZDHHC23B*
**Protein phosphatase**			*CDC25B*, *PLPP3*
**Pyrophosphatase**		*PPA1B*	
**Scaffold/adaptor protein**			*RB1CC1*
**Secondary carrier transporter**	*SLC2A1B*		
**Transferase**	*CHST11*	*CHST15*, *GSTA1*	
**Translation factor**	*PDCD4B*		
**Translation elongation factor**	*TUFM*		
**Transmembrane signal receptor**			*LEPROTL1*
**Transporter**			*AQP7*
**Ubiquitin-protein ligase**	*MOCS3*		
**Uncharacterized**	*CUNH2ORF69*, ***INAVAB***, ***PAGR1***, *PRCC*, *RHPN1*, *RNF146*, *TOMM6*, *TTC13*, *ZNF512B*	***INAVAB***, *FARP1*, ***PAGR1***, *RUNDC3AA*	*LRPPRC*, *MANEA*, *SSR4*

Note.—This table shows the set of genes that were determined by aBSREL, BUSTED, or RELAX to have come under changed selective pressure in the icefishes compared with the red-blooded cryonotothenioids. Genes are characterized according to their Panther protein classification (Thomas et al. 2003), serving to highlight their diverse range of functions. Genes that were identified as experiencing positive selective pressure by both aBSREL and BUSTED are bolded in the table.

Finally, the ability to discriminate selective change on each orthogroup is based on the success of the homology-based approach to pruning paralog contamination used in this study. To evaluate this approach, we compared our findings to an alternate set of orthogroups determined as described in Birkeland et al. (2020). As detailed in the Supplementary material, this continued to show the same broad trends in the number of genes under changed selection with cold adaptation and loss of hemoproteins. Although the Birkeland et al. (2020) approach showed a reduced ability to identify change in functional classes of genes, it did continue to show significant signatures of positive selection acting on a core group of gene regulatory factors as determined by both BUSTED and aBSREL, consistent with what we have found using our primary approach to paralog pruning.

## Discussion

The radiation of the cryonotothenioids in the isolation of the frigid Southern Ocean would have exposed these fishes to distinct selective pressures compared with their cold-temperate precursor. However, the extent to which these shifts in selective pressure are reflected across the pool of protein-coding genes has remained unclear. In this study, we found that evolution in the chronic cold is correlated with signatures of positive selective pressure acting heavily on proteins controlling gene expression along with basic cellular functions, suggesting key roles in cold adaptation in cryonotothenioids. This contrasts with the signatures of relaxed selective pressure, where a far narrower biological impact was resolved. Finally, in the icefishes, a small set of protein-coding genes were found to experience a change in selective pressure following the loss of hemoproteins despite widespread anatomical and physiological changes we see in this cryonotothenioid family.

### Genes under Positive Selective Pressure in the Red-Blooded Cryonotothenioids

The freezing temperatures of the Southern Ocean present clear challenges to life. Although the most immediate threat these fishes face comes from inoculative freezing, the extremely low water temperatures broadly impact biological processes. The set of genes showing signatures of positive selective pressure is suggestive of which biological functions may have been involved in polar adaptation in our focal group.

The largest signature of change comes from a collection of genes with roles controlling gene expression. These include varied gene regulatory factors: transcription factors, transcription activators, transcription repressors, histone-modifying enzymes, and signaling proteins. Among protein-coding genes, changes to such gene regulatory factors can have particularly wide-ranging impacts, as this can influence expression across the gene regulatory factor's target genes as well (Tirosh et al. 2009; Nowick et al. 2011; Perdomo-Sabogal et al. 2014). Prior work has shown that evolutionary change in gene regulatory factors regularly occurs and that such change may be a driving force behind species diversification and evolutionary innovation (Wagner and Lynch 2008; Nowick et al. 2013; Perdomo-Sabogal et al. 2014).

In terms of how this may relate to the evolution of the cryonotothenioids, today these fishes show a highly modified transcriptional program compared with temperate fishes (Chen et al. 2008). This includes increased expression of genes with biological roles that would mitigate many cold induced stresses, with roles in protein biosynthesis, protein folding and degradation, lipid metabolism, antioxidation, antiapoptosis, innate immunity, and choriongenesis, among others. Given capacity for widespread influence on the expression of downstream genes, changes to broadly acting gene regulatory factors such as those found under positive selection may thus have played a role in creating the cold-adapted transcriptome now seen among the cryonotothenioids.

In addition to changes that may impact the types of genes that are expressed, the set of genes under positive selection included several involved in the process of gene expression itself. The genes showing signatures of positive selection include those mediating RNA polymerase activity (*MED23*, *TCEA1*, and *UTP15*) and mRNA processing (*THOC5*). Prior studies have suggested that nascent protein synthesis (Pace and Manahan 2007) and their folding (Place and Hofmann 2005) are challenges to life in freezing polar waters. Our findings may suggest that the efficient transcription came under selection with adaptation to low temperature as well. Transcription has been shown to exhibit reduced efficiency and increased errors as an organism moves from physiological temperatures (Meyerovich et al. 2010). Adaptation to cold temperatures could thus exert selective pressure on the machinery of transcription to ensure continued accurate gene expression with the transition from temperate to polar conditions.

Alongside changes related to the pattern and process of gene expression, the set of genes under positive selection suggests compensatory change to adapt to the rate-limiting effects of low temperature on biological systems. This change is first seen through impacts to genes with essential roles in the cell cycle. A critical constraint on an organism's viable temperature range is the temperature range over which cell division can occur (Begasse et al. 2015), as this dictates the temperature range that growth and development can occur. The cryonotothenioids show change to several components of the machinery of cellular replication (*GNAI1*, *KIF22*, *NUF2*, and *RTF2*), suggesting adaptative change has acted on this toolkit to enable a basic activity of life to continue at freezing temperature.

In addition to challenges to the functioning of the cell cycle from the cold, the rate-limiting effects of low temperature present a further problem. However, past comparative studies have shown that adaptation can compensate for the rate-limiting effect of the cold on growth rates (Clarke 2003), and this seems to extend to the cell cycle itself. Investigation of cell cycle progression in the cryonotothenioid *Harpagifer antarcticus* found this to progress at a faster rate at 0 °C than in its sub-Antarctic congener *Harpagifer bispinis* at 5 °C suggesting cold compensation in the Antarctic species (Brodeur et al. 2003). Here, signatures of positive selective pressure were seen among genes related to cell cycle progression, including genes with a direct role in its regulation and progression (*CC14A*, *GNL2*, *DRG1*, *PDC6I*, and *PRCC*). This could represent parts of the genetic change that allow the cell cycle to progress at a biologically relevant rate at the low temperatures of the Southern Ocean.

Finally, we see signatures of positive selection among genes associated with cell adhesion. These included components of the junctions themselves (*CADH1*, *DSC2L*, and *EPCAM*) that link intercellular connections to the cytoskeleton (*CTNA1*) and that regulate cell junction organization (*TMM47*). The ability of cells to adhere to one another is strongly impacted by temperature, with a reduced capacity for adhesion as temperatures drop as cell–cell linkages lose the ability to organize and maintain cohesion (Attramadal 1975; Rico et al. 2010; Zieger et al. 2011). Thus, the capacity to organize and maintain adhesion may have come under selective pressure in the cryonotothenioids in order to maintain the integrity of tissues in freezing waters.

### Genes under Relaxed Selective Pressure in the Red-Blooded Cryonotothenioids

The Southern Ocean is remarkable for the chronic nature of its cold-water temperatures as much as the severity of this cold. Along with these stable low water temperatures, the surface waters of the Southern Ocean are oxygen rich as the oxygen carrying capacity of water increases with decreasing temperature (DeVries and Steffensen 2005). Wave-driven mixing action then ensures that even habitats at depth are saturated with oxygen across much of the Antarctic's continental shelf. The onset of these cold and oxygen-rich conditions would have relaxed selective pressure for the ancestor of the cryonotothenioids coming from an environment that was far more variable in both. Here, enrichment analysis on the set of genes putatively under changed selective pressure suggests that transition from a temperate to a chronic cold environment has left an impact on the genetic toolkit of the cryonotothenioids, in particular through change to genes related to mitochondrial function (fig. 3*B*; supplementary table S10, Supplementary Material online).

These genes span a range of roles within the mitochondria, including several components of the electron transport chain (*SDHA*, *NDUA8*, and *UQCRC2*), genes essential to mitochondrial function (*FASTKD1*, *MCAT*, *MPC2b*, *NNT*, *PRODHA*, and *SLC25A44*), and genes associated with biosynthesis within the mitochondria (*METTL17*, *MRPL41*, *MRPL53*, *MRPS17*, *MRPS22*, *MRPS30*, *TACO1*, and *TEFM*) or of essential mitochondrial proteins (*COA7*, *DDX28*, and *TOMM22*). Recent work analyzing the types of selection acting on the protein-coding genes in the notothenioid's mitochondrial genome by Papetti et al. (2021) concluded that a relaxation of selection was active early in the evolution of the cryonotothenioids, perhaps driven by the initial onset of cooling conditions. The results of this study suggest that the pattern of selective change may extend across the broader set of genes related to mitochondrial function, not just those restricted to the mitochondrial genome itself.

As for the source of changed selective pressure, one explanation may be the increased oxygen availability as the Southern Ocean cooled. Variable oxygen levels are a challenge for living things that rely on aerobic respiration given oxygen's role as the terminal electron acceptor in the mitochondria's electron transport chain. Cells of aerobic organisms maintain a toolkit to ensure oxygen homeostasis to ensure a constant rate of respiratory activity even as oxygen levels may vary (Longmuir 1957; Wilson et al. 1979). The onset of persistent high oxygen levels in the Southern Ocean could thus have reduced the necessity of maintaining such flexibility in mitochondrial function.

Besides oxygen availability, the signature of relaxed selection observed on mitochondrial genes may also be a consequence of the temperature stability of the Southern Ocean's water temperatures. Study of mitochondrial capacities in Antarctic notothenioids by Mark et al. (2012) showed that the mitochondrial capacities of these Antarctic fishes showed thermal limits that were lower than those of temperate species. Relaxed selective pressure broadly across the genetic toolkit of the mitochondria thus may alternatively reflect the lack of demand to maintain mitochondrial function at temperatures that are no longer encountered in cold Antarctic waters.

### Genes under Changed Selective Pressure in the Icefishes

Among the highly cold-adapted cryonotothenioid fishes, the species of the family Channichthyidae are remarkable for their lack of hemoproteins, but has this loss led to extensive change among the pool of protein-coding genes? Cold adaptation, which was explored using the red-blooded cryonotothenioids, is a very broad trait with widespread expected consequences across biological systems, and therefore we expect there should be many genes involved; in contrast, the loss of hemoproteins is a far more specific trait, so we expected to find fewer genes under selection. Our results tested this hypothesis—we identified a diverse suite of genes under cold-adapted selection and a few specific genes under hemoprotein-less selection.

Although relatively few genes showed signatures of changed selective pressure in the icefishes when compared with the red-blooded cryonotothenioids, this set of icefish genes under changed selective pressure was diverse (table 1). The small number of genes under positive or relaxed selective pressure did not result in significant enrichment of GO terms after correcting for multiple hypothesis testing, but interrogating this gene list, several of the genes under positive selection have roles that correspond to phenotypic change observed in the icefishes. These included genes with roles in cardiac morphogenesis, myogenesis, and vascular development (*MEF2C* and *NIPLB*), change that is correlated with the highly modified cardiovascular system seen in the icefishes that allows the larger blood volume necessary to transport oxygen in blood plasma without the presence of red blood cells (Sidell and O'Brien 2006). Interestingly, Bargelloni et al. (2019) found that several genes of the myocyte enhancer factor (MEF family), including *MEF2C*, are largely upregulated in the icefish and these may play an important role in the larger mitochondrial density seen in icefish cells. Similarly, several genes with roles in energy metabolism (*F16P1*, *GTR1*, and *PAQR1*) correspond to increases in mitochondrial size and density within icefish cells, and perhaps the need to keep cellular metabolism fueled in these fishes as oxygen availability is more limited. Finally, selection was seen in genes related to cartilage biosynthesis (*CHSTB*) and neural patterning (*IRX3*) both of which would likely be tied to the continued modification of skeletal and skull shape than what is seen in the remaining cryonotothenioids.

Besides the more limited scope of the comparison compared with cold adaptation, are there other reasons why we might observe such muted signatures of genetic change among protein-coding genes following hemoprotein loss? One possibility is that the relatively recent divergence of the icefishes has not allowed sufficient time to see widespread changes in protein-coding genes even if selective pressures have shifted. Alternatively, Bargelloni et al. (2019) suggested that the loss of hemoglobins has been accompanied by change in mechanisms like gene silencing or gene expression regulation more than change to protein-coding sequences. This would correspond to recent findings by Daane et al. (2020) and Bista et al. (2022) where investigation of genetic regions controlling erythropoiesis in the icefishes found clearer signatures of drift acting on the CNEs that control gene behavior rather than the coding regions themselves. Finally, prior work has also shown that the oxygen-rich Southern Ocean environment relaxed selective pressure on oxygen carriers before hemoprotein loss. This is observed in the reduction in hemoglobin isoform complement among the red-blooded cryonotothenioids (Eastman 1993) and in the attenuation of regulatory elements upstream of hemoglobin (Lau et al. 2012) in both the red-blooded cryonotothenioids and the icefishes (Daane et al. 2020). As a result, the icefishes may simply reflect an extreme result of the same selective forces that are acting across the cryonotothenioids as a whole.

### Conclusions

The genomes of the cryonotothenioid fishes have been changed during their evolution in the isolation of the Southern Ocean as seen among their suite of protein-coding genes. The observed changes reflect not only the abiotic challenges of life in a cold and oxygen-rich environment but also the stability of the region's waters.

The influence of positive selective pressure was most strongly felt on proteins with roles in gene expression, suggesting one route through which the cryonotothenioid transcriptomic profile was retooled for life in the cold. Further change observed in genes with roles in the machinery of transcription, cell cycle progression, and in cell adhesion, pointing to biological challenges for life in a freezing ocean. Contrasting with the influence of positive selection, the relaxation of selective pressure appears to have exerted a narrower influence. This seems to have driven change to a diverse group of genes related to mitochondrial function, perhaps due to the reduced challenges of carrying out aerobic metabolism given the cold-stable temperatures and high oxygen levels found in Southern Ocean waters. Finally, in icefishes, the loss of hemoglobin has resulted in far fewer detected signatures of change in the sequence of protein-coding genes, perhaps due to the smaller number of possible target genes compared with cold adaptation or due to the alternative genetic and physiological mechanisms that may have compensated for this unique loss.

In evaluating these changes, one note of caution is that we are limited by the number of orthologous relationships that we could identify among the protein-coding sequences of the target species. It is likely that further biological systems under positive and relaxed selective pressures have yet been discovered in this group as a fuller view of protein-coding genes becomes available.

Ultimately though, cryonotothenioid fishes have been exposed to long and intense selective pressure for survival in freezing waters and now show widespread change when compared with temperate fishes including long development and generation times, deferred maturity, and extended life spans (Peck 2016). The genetic change we identified in the cryonotothenioids since the onset of freezing conditions may further suggest a genetic specialization to life in the cold that would leave them particularly ill-equipped to deal with a warming world. In light of these changes, it seems unlikely that any reverse adaptation might be possible in the short term of the predicted climatic alteration occurring in Antarctica (Masson-Delmotte et al. 2021).

## Materials and Methods

### Genomic Resources, Sequence, Assembly, and Filtering

To investigate how the pool of protein-coding genes have been affected by the ecological and physiological transitions experienced during the radiation of the cryonotothenioids, public genomic and transcriptomic resources were compiled for the cryonotothenioids along with a relevant background set of temperate and tropical fishes. In total, protein-coding sequences were compared across 19 species including 7 red-blooded cryonotothenioids, 5 hemoglobin-lacking icefishes, and 7 temperate and tropical fishes. The full list of the species used in this study and accession numbers for the genomic and transcriptomic resources used in the analysis can be found in the supplementary material (supplementary table S1, Supplementary Material online). Of these, genome-derived predicted protein-coding gene sequences were publicly available at the time of this study for only three red-blooded species: *D. mawsoni* Norman, 1937 (Nototheniidae; Chen et al. 2019); *Notothenia coriiceps* Richardson, 1844 (Nototheniidae; Shin et al. 2014); and *Parachaenichthys charcoti* Vaillant, 1906 (Bathydraconidae; Ahn et al. 2017). To extend this limited data set, sequenced transcriptomic reads were downloaded for species where more than one tissue was available, including the three red-blooded species with available sequenced genomes (Bilyk et al. 2018; Bargelloni et al. 2019; Berthelot et al. 2019; Kim et al. 2019; Song et al. 2019).

To provide a baseline for evaluating changes in selective pressure in the cryonotothenioids, predicted peptides and coding domain sequences (CDS) were compiled for a background set of temperate and tropical fishes. These included two extant temperate notothenioid fishes that diverged prior to the adaptation to polar conditions in the cryonotothenioids, the Patagonian blenny *Eleginops maclovinus* Cuvier, 1830 (Eleginopidae; Chen et al. 2019) and *Cottoperca gobio* Günther, 1861 (Bovichtidae; Bista et al. 2020). The remaining species were selected from the teleost fishes available from Ensembl (Yates et al. 2020), with the aim to minimize phylogenetic distance, avoiding obligatory freshwater species, avoiding species that inhabit freezing environments, and minimizing nested taxa. Species native to freezing habitats were avoided in the background set as this would likely obscure signatures of change among biological systems resulting from adaptation to freezing conditions among the cryonotothenioids.

The transcriptomic reads were assembled for all of the cryonotothenioids. The reads were first cleaned with Fastp v0.20.1 (Chen et al. 2018) and then assembled with Trinity v. 2.6.5 (Grabherr et al. 2011) using default parameters. Assemblies then went through preliminary filtering to remove redundancy with CD-hit v. 4.8.1 (Fu et al. 2012) and by removing contigs with <1 transcripts per million (TPM) read coverage based on the transcriptome's original sequenced reads. Predicted peptides and their corresponding CDS were then determined for the transcriptomes using TransDecoder v. 5.5.0 (Haas 2021). These predicted peptides were subjected to a final round of filtering meant to reduce much of the redundancy present in each species’ transcriptome. Predicted peptides were mapped against the Swissprot database using BlastP v2.10.1, retaining only the best match for each Swissprot accession number as determined by *e* value.

Finally, the relative completeness of species’ genome- or transcriptome-derived set of predicted peptides was evaluated using BUSCO v. 4 (Simão et al. 2015). BUSCO provides a measure of the assembly's gene completeness by quantifying the presence of conserved single copy orthologous genes. In addition to providing a general measure of quality for each species’ gene set, BUSCO scores were used to choose between genomic- and transcriptomic-derived predicted peptides and CDS when both were available for a given species. The transcriptomic assemblies were generally found to show higher levels of complete genes likely reflecting the oversampling that is inherent to transcriptomic sequencing, though this came at the expense of greater redundancy which had to be filtered.

### Phylogenetic Reconstruction

To provide a phylogenetic frame of reference for later evolutionary hypothesis testing, we reconstructed the relationship among the 19 investigated species. This phylogeny was constructed using 2 mitochondrial (*16S* and *ND2*) and 11 nuclear genes (*MYH6*, *PKD1*, *SH3PX3*, *HECW2*, *SSRP1*, *PPM1D*, *RPS71*, *TBR1*, *PTR*, *RHO*, and *ZIC1*). Accession numbers for the genes used in this analysis are provided in supplementary table S2, Supplementary Material online.

The sequences of each gene were aligned using MUSCLE v. 3.8.31 (Edgar 2004), and the best-fit nucleotide substitution model of each gene was determined using the Akaike information criterion (AIC) in ModelTest-NG v0.1.6 (Darriba et al. 2020). The aligned sequences were concatenated and partitioned according to their best-fit models (GTR + I + gamma, mitochondrial: ND2 and 16S; GTR + I + gamma: MYH6 and PKD1; GTR + gamma: SH3PX3, HECW2, and SSRP1; GTR: PPM1D; HKY + gamma: RPS71, TBR1, PTR, and RHO; HKY + I: ZIC1). These substitution models were then implemented using MrBayes v3.2.6 (Ronquist et al. 2012) to carry out Bayesian phylogenetic analyses. The Markov chain Monte Carlo (MCMC) simulation was run for 100 million generations with four chains and sampled every 100 generations. MCMC convergence was assessed using the standard deviation of clade frequencies and potential scale reduction factor, and the first 25% of sampled trees were discarded as burn-in. Clade support was evaluated using posterior probabilities for nodes retained in the 50% majority rule consensus tree.

### Orthogroup Inference, Paralog Pruning and Building the MSAs

Orthogroups were inferred across species from their filtered set of predicted peptides using OrthoFinder v. 2.5.1 (Emms and Kelly 2019). The resulting orthogroups were then filtered to remove potential paralog contamination using a two-step process. First, PhyloTreePruner v. 1.0 (Kocot et al. 2013) was used to isolate the largest monophyletic subtree from the gene trees generated by FastTree 2 (Price et al. 2010) produced for the contigs within each orthogroup. Second, the subtree was collapsed to a set of species-specific putative orthologs using BlastP against the *D. mawsoni* predicted peptide set. In this last step, contigs were only considered to have an orthologous relationship if they were best Blast hits, as determined by *e* value, to the same *D. mawsoni* predicted peptide. If more than one contig from a species matched the same *D. mawsoni* predicted peptide, then only the best Blast hit, as determined by *e* value, was retained. If more than one transcript was present from *D. mawsoni* in the orthogroup, the orthogroup was subset for each *D. mawsoni* transcript, and contigs from other species were assigned only to the *D. mawsoni* transcript with the best Blast hit. Finally, only filtered orthogroups with representatives of all 19 target species were retained for constructing the multiple sequence alignments (MSAs). To evaluate our approach to paralog pruning, we generated an additional set of filtered orthogroups using the approach described in Birkeland et al. (2020), which is presented in the Supplementary material.

MSAs were then generated for each retained orthogroup. The CDS for each orthogroup's predicted peptides were codon aligned with GUIDANCE2 (Sela et al. 2015) using Prank v.140603 (Löytynoja 2014), running 25 pseudo replicates per alignment. The alignments were then trimmed to remove any missing sites, and only alignments with all species and a minimum final length of 300 nt were retained for evolutionary hypothesis testing.

### Identifying Orthogroups under Changed Selective Pressure

Orthogroups experiencing positive selective pressure were identified using the phylogeny-based strategy implemented in the BUSTED (Murrell et al. 2015) and the aBSREL (Smith et al. 2015) methods in HyPhy (Kosakovsky Pond et al. 2020). The contrasting set of orthogroups experiencing a relaxation pressure was identified using the RELAX method in HyPhy (Wertheim et al. 2015).

The orthogroups were first used to compare protein-coding genes in the red-blooded cryonotothenioids as the foreground against the background set of temperate and tropical fishes to identify the set of orthologous genes that have come under changed selective pressure during the cryonotothenioid radiation in the frigid Southern Ocean. Similarly, the icefishes were tested as the foreground against a background of red-blooded cryonotothenioids to identify the set of orthogroups that have come under changed selective pressure specific to the loss of hemoproteins. A FDR-adjusted *P*-value threshold of 0.1 was used in all tests to identify those orthogroups showing a significant change in selective pressure from the background set.

### Testing for Functional Enrichment

GO enrichment analysis was used to place the impacts of positive and relaxed selective pressures into biological context. The *D. mawsoni* predicted peptide set was annotated for GO terms using the Orthologous Matrix fast mapping utility (OMA browser; Altenhoff et al. 2021). The resulting GO annotations were then used to generate a slimmed set of GO terms for *D. mawsoni* using Blast2GO (Conesa et al. 2005). GO slim terms were then applied across an orthogroup if it contained the original annotated *D. mawsoni* predicted peptide.

TopGO v. 2.44 (Alexa and Rahnenfuhrer 2022) was used to test for functional enrichment among the isolated sets of genes under positive diversifying or relaxed selective pressure within the biological process (BP), molecular function (MF), and cellular component (CC) ontologies from the GO-slim annotation sets. Enrichment was tested using the sets of significant orthogroups identified by BUSTED, aBSREL, and RELAX, against a background set of 3,452 orthogroups with representatives from all 19 species originally used in the analysis. Fisher's exact test was then used to identify enriched terms using an FDR adjusted *P*-value threshold of 0.1, both on the results of the individual tests, on the intersected set of genes where both BUSTED and aBSREL agreed on signatures of positive selection, and on the combined set of genes identified under positive selection by either BUSTED or aBSREL. The identified set of significant GO terms was then visualized using the EnrichmentMap (Merico et al. 2010) plug-in of Cytoscape network visualization software v. 3.8.2 (Shannon et al. 2003).

## Supplementary Material

evad049_Supplementary_Data

## Data Availability

All genomic and read data used in this study were downloaded from publicly available repositories as detailed in the Supplementary material. The scripts used in this study are available through the GitHub repository: https://github.com/TheOneTrueKevin/2023-Positive-and-Relaxed-Selective-Pressure-on-Cryonotothenioid-Fishes.
