# Supplementary material for: Positive and Relaxed Selective Pressures Have Both Strongly Influenced the Evolution of Cryonotothenioid Fishes during Their Radiation in the Freezing Southern Ocean
Source: Genome Biol Evol. 2023 Mar 23;15(4):evad049. doi: 10.1093/gbe/evad049 (PMC10078794; doi:10.1093/gbe/evad049)
Supplement: evad049_Supplementary_Data [file evad049_supplementary_data.zip › S Material 2023-03-02.docx]

**Supplementary Material # 1**

**Verification of enriched GO terms using an alternate approach to paralog pruning**

***Overview & Methods***

To evaluate our approach to paralog pruning we compared our findings to the alternate approach used by Birkeland et al (2020). Here, orthogroups were inferred across species from the filtered set of predicted peptides using OrthoFinder v. 2.5.1 (Emms and Kelly, 2019). Orthologs were separately determined for two groups of comparisons: the red-blooded cryonotothenioids against temperate fishes, and the icefishes (channichthyids) against the red-blooded cryonotothenioids. Identifying distinct sets of orthogroups was deemed necessary due to the lower overall number of orthogroups identified using this protein distance approach, and the split set allowed us to maximize the gene sets available to test for significant change in functional categories of genes. This was especially important for the Icefish comparison to reduce the risk that a lack of genes detected under selection was due to a small pool of tested genes.

The resulting orthogroups were then filtered to remove potential paralog contamination following the approach described in Birkeland et al. (2020) as described below. In brief, MAFFT protein sequence alignments for each orthogroup were used to determine the Kimura protein distance (Kimura 1983) between all of the orthogroup’s contigs using the distmat program available through the EMBOSS suite of bioinformatics software tools (Rice et al. 2000). For both sets of comparisons, orthogroups were then condensed to just one gene per species based on the smallest genetic distance to the transcript for the red-blooded cryonotothenioid *D. mawsoni*. If more than one transcript was present from *D. mawsoni* in the orthogroup, than the orthogroup was subset for each *D. mawsoni* transcript, and contigs from other species were assigned only to the *D. mawsoni* transcript with the smallest evolutionary distance. The resulting orthogroups contained only one transcript per species and were filtered to retain only those that contained representatives of all target species for the comparison.

Multiple sequence alignments were then generated for each retained orthogroup. The CDS for each orthogroup’s predicted peptides were codon aligned with GUIDANCE2 (Sela et al, 2015) using Prank v.140603 (Löytynoja, 2014), running 25 pseudo replicates per alignment. The alignments were then trimmed to remove any missing sites, and only alignments with all species and a minimum final length of 300nt were retained for evolutionary hypothesis testing.Testing for orthogroups showing signatures of changed selective pressure were then carried out for the red-blooded cryonotothenioid and icefish comparisons as described in the main text.

***Results & Discussion***

In total, 3,513 orthogroups for the red-blooded cryonotothenioid and temperate fish comparison, as well as 4,700 orthogroups for the icefish and red-blooded cryonotothenioid comparison. Tests for selective pressure using an FDR 0. 1 p value threshold then identified:

**Genes under positive selection: Red-blooded cryonotothenioids vs temperate fishes**

| BUSTED only | Both BUSTED & aBSREL | aBSREL only |
| --- | --- | --- |
| 35 | 40 | 75 |

**Genes under intensified or relaxed selective pressure by: Red-blooded cryonotothenioids vs temperate fishes**

| RELAX K > 1 (intensification) | RELAX K < 1 (relaxation) |
| --- | --- |
| 71 | 119 |

**Genes under positive selection: Icefish vs red-blooded cryonotothenioids**

| BUSTED only | Both BUSTED & aBSREL | aBSREL only |
| --- | --- | --- |
| 8 | 4 | 32 |

**Genes under intensified or relaxed selective pressure by: Icefish vs red-blooded cryonotothenioids**

| RELAX K > 1 (intensification) | RELAX K < 1 (relaxation) |
| --- | --- |
| 7 | 7 |

Out of these gene sets, only the set of 40 genes identified by both BUSTED & aBSREL as being under positive selection resulted in significant enrichment of GO terms

| **GO.ID** | **Term** | **Significant** | **P value** | **FDR** |
| --- | --- | --- | --- | --- |
| GO:0006351 | transcription, DNA-templated | 12 | 0.00054 | 0.012953 |
| GO:0018130 | heterocycle biosynthetic process | 12 | 0.00054 | 0.012953 |
| GO:0019438 | aromatic compound biosynthetic process | 12 | 0.00054 | 0.012953 |
| GO:0032774 | RNA biosynthetic process | 12 | 0.00054 | 0.012953 |
| GO:0034654 | nucleobase-containing compound biosynthe... | 12 | 0.00054 | 0.012953 |
| GO:0097659 | nucleic acid-templated transcription | 12 | 0.00054 | 0.012953 |
| GO:1901362 | organic cyclic compound biosynthetic pro... | 12 | 0.00054 | 0.012953 |
| GO:0044271 | cellular nitrogen compound biosynthetic ... | 12 | 0.00063 | 0.012953 |
| GO:0065007 | biological regulation | 12 | 0.00114 | 0.012953 |
| GO:0006355 | regulation of transcription, DNA-templat... | 11 | 0.00128 | 0.012953 |
| GO:1903506 | regulation of nucleic acid-templated tra... | 11 | 0.00128 | 0.012953 |
| GO:2001141 | regulation of RNA biosynthetic process | 11 | 0.00128 | 0.012953 |
| GO:0009889 | regulation of biosynthetic process | 11 | 0.00131 | 0.012953 |
| GO:0010556 | regulation of macromolecule biosynthetic... | 11 | 0.00131 | 0.012953 |
| GO:0019219 | regulation of nucleobase-containing comp... | 11 | 0.00131 | 0.012953 |
| GO:0031326 | regulation of cellular biosynthetic proc... | 11 | 0.00131 | 0.012953 |
| GO:0051252 | regulation of RNA metabolic process | 11 | 0.00131 | 0.012953 |
| GO:2000112 | regulation of cellular macromolecule bio... | 11 | 0.00131 | 0.012953 |
| GO:0031323 | regulation of cellular metabolic process | 11 | 0.00134 | 0.012953 |
| GO:0051171 | regulation of nitrogen compound metaboli... | 11 | 0.00134 | 0.012953 |
| GO:0080090 | regulation of primary metabolic process | 11 | 0.00134 | 0.012953 |
| GO:0050794 | regulation of cellular process | 11 | 0.00143 | 0.013039 |
| GO:0010467 | gene expression | 12 | 0.00154 | 0.013039 |
| GO:0010468 | regulation of gene expression | 11 | 0.00167 | 0.013039 |
| GO:0019222 | regulation of metabolic process | 11 | 0.00167 | 0.013039 |
| GO:0060255 | regulation of macromolecule metabolic pr... | 11 | 0.00167 | 0.013039 |
| GO:0050789 | regulation of biological process | 11 | 0.00178 | 0.013226 |
| GO:0016070 | RNA metabolic process | 13 | 0.00203 | 0.013226 |
| GO:0009058 | biosynthetic process | 12 | 0.00215 | 0.013226 |
| GO:0009059 | macromolecule biosynthetic process | 12 | 0.00215 | 0.013226 |
| GO:0034645 | cellular macromolecule biosynthetic proc... | 12 | 0.00215 | 0.013226 |
| GO:0044249 | cellular biosynthetic process | 12 | 0.00215 | 0.013226 |
| GO:1901576 | organic substance biosynthetic process | 12 | 0.00215 | 0.013226 |
| GO:0006139 | nucleobase-containing compound metabolic... | 14 | 0.00282 | 0.015472 |
| GO:0006725 | cellular aromatic compound metabolic pro... | 14 | 0.00282 | 0.015472 |
| GO:0046483 | heterocycle metabolic process | 14 | 0.00282 | 0.015472 |
| GO:1901360 | organic cyclic compound metabolic proces... | 14 | 0.00282 | 0.015472 |
| GO:0034641 | cellular nitrogen compound metabolic pro... | 14 | 0.00315 | 0.016828 |
| GO:0044260 | cellular macromolecule metabolic process | 12 | 0.00351 | 0.01827 |
| GO:0090304 | nucleic acid metabolic process | 13 | 0.00375 | 0.019031 |
| GO:0009987 | cellular process | 25 | 0.01056 | 0.052285 |
| GO:0006807 | nitrogen compound metabolic process | 19 | 0.01759 | 0.085018 |
| GO:0043170 | macromolecule metabolic process | 18 | 0.02094 | 0.098856 |

The rows in red show conserved GO terms with the intersection set in the original analysis and highlight a consistent influence of positive selective pressure across classes of genes with key roles controlling gene expression. This is showing a consistent signature of the functional class of gene under positive selection.

At the level of the individual gene, this set of 40 genes includes many gene regulatory factors, though there are differences from the set of genes identified in the original analysis.

| ***D. mawsoni* contig ID** | **Gene ID** | **Gene Name** |
| --- | --- | --- |
|  |  |  |
| Dissostichus_mawsoni_GLEAN_10014478 | ephb4a | eph receptor B4a (ephb4a), mRNA |
| Dissostichus_mawsoni_GLEAN_10009496 | celf3b | CUGBP Elav-like family member 3-B |
| Dissostichus_mawsoni_GLEAN_10013206 | fmnl3 | formin-like 3 (fmnl3) |
| Dissostichus_mawsoni_GLEAN_10008622 | sept7a | septin 7-like |
| Dissostichus_mawsoni_GLEAN_10014834 | abhd17ab | abhydrolase domain containing 17A, depalmitoylase b (abhd17ab) |
| Dissostichus_mawsoni_GLEAN_10021685 | sh3gl2a | endophilin-A1-like |
| Dissostichus_mawsoni_GLEAN_10015657 | baiap2l1b | brain-specific angiogenesis inhibitor 1-associated protein 2-like |
| Dissostichus_mawsoni_GLEAN_10014068 | trpv1 | transient receptor potential cation channel subfamily V member 1 |
| Dissostichus_mawsoni_GLEAN_10016063 | mef2aa | myocyte enhancer factor 2aa (mef2aa), transcript variant X7, mRNA |
| Dissostichus_mawsoni_GLEAN_10011043 | mapk1 | mitogen-activated protein kinase 1 (mapk1), mRNA |
| Dissostichus_mawsoni_GLEAN_10015778 | raf1a | Raf-1 proto-oncogene, serine/threonine kinase a (raf1a) |
| Dissostichus_mawsoni_GLEAN_10021485 | tgfb3 | transforming growth factor beta 3 (tgfb3) |
| Dissostichus_mawsoni_GLEAN_10010824 | stac3 | SH3 and cysteine-rich domain-containing protein 3-like (stac3) |
| Dissostichus_mawsoni_GLEAN_10009774 | ogna | osteoglycin, paralog a (ogna)... listed as mimecan-like in some |
| Dissostichus_mawsoni_GLEAN_10018274 | irf3 | interferon regulatory factor 3 (irf3) |
| Dissostichus_mawsoni_GLEAN_10018182 | med24 | mediator complex subunit 24 (med24) |
| Dissostichus_mawsoni_GLEAN_10010870 | pdcd4 | programmed cell death 4 (pdcd4) |
| Dissostichus_mawsoni_GLEAN_10011621 | zbtb7a | zinc finger and BTB domain containing 7a (zbtb7a) |
| Dissostichus_mawsoni_GLEAN_10013341 | dab2 | DAB adaptor protein 2 (dab2) |
| Dissostichus_mawsoni_GLEAN_10016301 | plpp3 | phospholipid phosphatase 3 (plpp3) |
| Dissostichus_mawsoni_GLEAN_10010290 | cdca8 | cell division cycle associated 8 (cdca8), mRNA |
| Dissostichus_mawsoni_GLEAN_10005573 | usf1 | upstream transcription factor 1 (usf1) |
| Dissostichus_mawsoni_GLEAN_10009214 | jazf1b | juxtaposed with another zinc finger protein 1 (LOC101061040), mRNA |
| Dissostichus_mawsoni_GLEAN_10015814 | ogg1 | 8-oxoguanine DNA glycosylase (ogg1), mRNA |
| Dissostichus_mawsoni_GLEAN_10013954 | nceh1a | neutral cholesterol ester hydrolase 1-like (LOC109987577), mRNA |
| Dissostichus_mawsoni_GLEAN_10010027 | tmem104 | Transmembrane Protein 104 |
| Dissostichus_mawsoni_GLEAN_10022213 | paxbp1 | PAX3 and PAX7 binding protein 1 (paxbp1), mRNA |
| Dissostichus_mawsoni_GLEAN_10020590 | ick-like | serine/threonine-protein kinase ICK-like (LOC117536745), mRNA |
| Dissostichus_mawsoni_GLEAN_10018727 | zdhhc23 | zinc finger DHHC-type containing 23 (zdhhc23), mRNA |
| Dissostichus_mawsoni_GLEAN_10019763 | apoa1 | apolipoprotein A-I-like (LOC115018293), mRNA |
| Dissostichus_mawsoni_GLEAN_10011324 | mpst | mercaptopyruvate sulfurtransferase (mpst), mRNA |
| Dissostichus_mawsoni_GLEAN_10012048 | fam53b | family with sequence similarity 53 member B (fam53b), mRNA |
| Dissostichus_mawsoni_GLEAN_10008925 | med30 | mediator complex subunit 30 (med30) |
| Dissostichus_mawsoni_GLEAN_10008741 | nop9 | NOP9 nucleolar protein (nop9), mRNA |
| Dissostichus_mawsoni_GLEAN_10012759 | mrps24 | mitochondrial ribosomal protein S24 (mrps24) |
| Dissostichus_mawsoni_GLEAN_10008527 | cdc42ep3 | CDC42 effector protein 3 (cdc42ep3), mRNA |
| Dissostichus_mawsoni_GLEAN_10004979 | xbp1 | X-box binding protein 1 (xbp1), mRNA |
| Dissostichus_mawsoni_GLEAN_10001716 | grap2a | GRB2 related adaptor protein 2a (grap2a), mRNA |
| Dissostichus_mawsoni_GLEAN_10004234 | cdr2-like | cerebellar degeneration-related protein 2-like |
| Dissostichus_mawsoni_GLEAN_10020700 | prdm4 | PR domain containing 4 (prdm4), mRNA |
|  |  |  |

Orthogroups are identified in the table above based on the *D. mawsoni* contig within the orthogroup. Table entries in red have matches among the set of genes identified under positive selection by both BUSTED and aBSREL in the original analysis, while entries in blue have matches among the larger set of genes identified under positive selection by either BUSTED or aBSREL.

The difference in orthogoups between the two approaches to paralog pruning are likely a result of BLAST and distmat defining a substantial number of distinct orthogroups. Further, using the intersection sets from both analyses to evaluate the validity of orthogroup determinations, we found greater levels of paralog contamination in orthogroups defined by the distmat approach compared to the BLAST based approach. This was evaluated by carrying out BLASTn on all the contigs in each orthogroup against NCBI’s NT database where the 40 orthogroups defined by the distmat approach included more orthogroups that showed incongruous gene names among contigs using the most informative BLAST results.

**REFERENCES**

Birkeland S, Lovisa A, Gustafsson S, Brysting AK, Brochmann C, Nowak MD (2020). Multiple Genetic Trajectories to Extreme Abiotic Stress Adaptation in Arctic Brassicaceae, Mol Biol Evol. 37(7) Pages 2052–2068

Emms DM, Kelly S (2019). OrthoFinder: phylogenetic orthology inference for comparative genomics. Genome Biology 20(1): 238.

Löytynoja A (2014). Phylogeny-aware alignment with PRANK. Methods in molecular biology (Clifton, NJ) 1079: 155-170.

Kimura M (1983). The neutral theory of molecular evolution. Cambridge: Cambridge.

Rice P, Longden I, Bleasby A. 2000. EMBOSS: The European Molecular Biology Open Software Suite. Trends Genet. 16(6):2–3.

Sela I, Ashkenazy H, Katoh K, Pupko T (2015). GUIDANCE2: accurate detection of unreliable alignment regions accounting for the uncertainty of multiple parameters. Nucleic Acids Res 43(W1): W7-14.
