# Supplementary material for: Positive and Relaxed Selective Pressures Have Both Strongly Influenced the Evolution of Cryonotothenioid Fishes during Their Radiation in the Freezing Southern Ocean
Source: Genome Biol Evol. 2023 Mar 23;15(4):evad049. doi: 10.1093/gbe/evad049 (PMC10078794; doi:10.1093/gbe/evad049)
Supplement: evad049_Supplementary_Data [file evad049_supplementary_data.zip › S Tables.20230227.docx]

**Positive and relaxed selective pressures have both strongly influenced the evolution of cryonotothenioid fishes during their radiation in the freezing Southern Ocean**

**Supplementary Tables**

**SECTION** **PAGE**

S Table 1 ………………………………………………………………………………………… 1

Genomic and transcriptomic resources used in this study

S Table 2 ………………………………………………………………………………………… 5

Genes used in Phylogenetic Reconstruction

S Table 3 ………………………………………………………………………………………… 7

Orthogroups identified as under both positive selective pressure and relaxed purifying selective pressure.

S Table 4 ………………………………………………………………………………………… 8

Enriched GO terms for orthogroups identified under positive selective pressure by only **aBSREL** in the red-blooded cryonotothenioids

S Table 5 ………………………………………………………………………………………… 10

Enriched GO terms for orthogroups identified under positive selective pressure by only **BUSTED** in the red-blooded cryonotothenioids

S Table 6 ………………………………………………………………………………………… 11

Enriched GO terms for orthogroups identified under positive selective pressure by **either (union) BUSTED or aBSREL** in the red-blooded cryonotothenioids

S Table 7 ………………………………………………………………………………………… 13

Enriched GO terms for orthogroups identified under positive selective pressure by **both (intersection) BUSTED and aBSREL** in the red-blooded cryonotothenioids

S Table 8 ………………………………………………………………………………………… 15

The 42 genes identified under positive selection by **both (intersection) BUSTED and aBSREL** in the red-blooded cryonotothenioids

S Table 9 ………………………………………………………………………………………… 17

The 160 genes identified under positive selection by **either (union) BUSTED and aBSREL** in the red-blooded cryonotothenioids

S Table 10 ………………………………………………………………………………………… 22

Enriched GO terms for orthogroups consistently identified under relaxed selective pressure by **RELAX** in the red-blooded cryonotothenioids when compared to a set of temperate and tropical fishes

S Table 11 ………………………………………………………………………………………… 23

The genes identified by RELAX as experiencing a significant relaxation of selective pressure in the red-blooded cryonotothenioids

S Table 12 ………………………………………………………………………………………… 29

The genes identified by RELAX as experiencing a significant relaxation of selective pressure in the red-blooded cryonotothenioids

S Table 13 ………………………………………………………………………………………… 40

The genes identified by RELAX as experiencing a significant relaxation of selective pressure in the red-blooded cryonotothenioids

S Table 14 ………………………………………………………………………………………… 42

The genes identified by RELAX as experiencing a significant relaxation of selective pressure in the red-blooded cryonotothenioids

S Table 15 ………………………………………………………………………………………… 43

The genes identified by RELAX as experiencing a significant relaxation of selective pressure in the red-blooded cryonotothenioids

**S Table 1:**

**Genomic & Transcriptome sequences used in the data analysis**

| **SPECIES** | **ACCESSION** | | **READS** | **TISSUE** |
| --- | --- | --- | --- | --- |
| *Eleginops maclovinus* (genome) ^x^ | doi:10.5524/102163 | | N/A | N/A |
| *Eleginops maclovinus* (transcriptome) | SRR5210465  (pool) | | 60,927,159 | Brain  Eye Cup  Gill  Head Kidney  Heart  Intestine  Liver  Lens  Spleen  Stomach |
| *Dissostichus mawsoni* (genome) ^x^ | doi:10.5524/102162 | | N/A | N/A |
| *Dissostichus mawsoni* (transcriptome) | SRR6794060 | | 17,065,548 | Brain |
|  | SRR6794071 | | 15,365,453 | Caudal Kidney |
|  | SRR6794062 | | 13,225,737 | Gill |
|  | SRR6794059 | | 15,639,916 | Head Kidney |
|  | SRR6794061 | | 16,644,938 | Liver |
|  | SRR6794069 | | 10,826,931 | Ovary |
|  | SRR6794065 | | 11,869,689 | Pelvic Girdle Bone |
|  | SRR6794063 | | 13,427,948 | Red Muscle |
|  | SRR6794066 | | 11,286,647 | Skin |
|  | SRR6794070 | | 11,579,054 | Spleen |
|  | SRR6794068 | | 13,532,039 | Small Intestine |
|  | SRR6794067 | | 18,596,244 | Stomach |
|  | SRR6794064 | | 12,810,566 | White Muscle |
| *Notothenia coriiceps* (genome) ^\|^ | GCF_000735185.1 | | N/A | N/A |
| *Notothenia coriiceps* (transcriptome) | SRR1015840 | | 18,989,174 | Blood (control) |
|  | SRR1015879 | | 19,417,020 | Blood (heat stress, 48 hours) |
|  | SRR1015880 | | 17,416,145 | Blood (heat stress, 48 hours) |
|  | SRR1015881 | | 18,871,617 | Blood (cold stress, 48 hours) |
|  | SRR1015882 | | 16,296,280 | Blood (cold stress, 48 hours) |
|  | SRR1015883 | | 19,987,559 | Brain (control) |
|  | SRR1015884 | | 16,808,128 | Brain (control) |
|  | SRR1015885 | | 18,235,881 | Brain (heat stress, 48 hours) |
|  | SRR1015886 | | 17,406,337 | Brain (heat stress, 48 hours) |
|  | SRR1015887 | | 16,598,395 | Brain (cold stress, 48 hours) |
|  | SRR1015888 | | 13,449,393 | Brain (cold stress, 48 hours) |
|  | SRR1015889 | | 17,491,754 | Blood (cold stress, 48 hours) |
|  | SRR1015890 | | 25,952,971 | Blood (cold stress, 48 hours) |
|  | SRR1015891 | 21,631,222 | | Skin (heat stress, 48 hours) |
|  | SRR1015892 | 16,914,027 | | Skin (heat stress, 48 hours) |
|  | SRR1015893 | 22,709,257 | | Skin (cold stress, 48 hours) |
|  | SRR1015894 | 15,845,995 | | Skin (cold stress, 48 hours) |
|  | SRR1015895 | 4,167,897 | | N/A |
|  | SRR1015896 | 25,000,000 | | N/A |
|  | SRR1015897 | 30,080,514 | | N/A |
|  | SRR1015898 | 30,000,000 | | N/A |
|  | SRR1015899 | 33,752,226 | | N/A |
|  | SRR1015900 | 2,538,814 | | N/A |
|  | SRR1015901 | 16,808,128 | | N/A |
| *Trematomus bernacchii* (transcriptome) | SRR7164563 | 1,306,383 | | Gonad, Male |
|  | SRR7164560 | 1,997,674 | | Head Kidney |
|  | SRR7164559 | 1,520,347 | | Heart |
|  | SRR7164558 | 2,354,231 | | Liver |
|  | SRR7164565 | 2,363,436 | | Muscle |
|  | SRR7164561 | 1,975,490 | | Skin |
|  | SRR7164570 | 1,943,699 | | Spleen |
|  | SRR7164564 | 461,588 | | Stomach |
| *Pagothenia borchgrevinki* (transcriptome) | SRR5210464  (pool) | 63,735,228 | | Brain  Eye Cup  Gill  Head Kidney  Heart  Intestine  Lens  Liver  Spleen  Stomach |
| *Harpagifer antarcticus* (transcriptome) † | ERR2587155 | 12,546,421 | | Brain |
|  | ERR2587156 | 20,170,567 | | Heart |
|  | ERR2587157 | 17,626,116 | | Kidney |
|  | ERR2587158 | 14,256,208 | | Liver |
|  | ERR2587159 | 11,757,830 | | Skin |
|  | ERR2587160 | 13,377,648 | | White Muscle |
| *Parachaenichthys charcoti* (genome) ^x^ | doi:10.5524/100321 | N/A | | N/A |
| *Parachaenichthys charcoti* (transcriptome)† | ERR2587183 | 20,190,257 | | Blood |
|  | ERR2587164 | 15,799,167 | | Brain |
|  | ERR2587166 | 20,526,312 | | Head kidney |
|  | ERR2587169 | 15,994,258 | | Liver |
|  | ERR2587171 | 11,340,950 | | Ovary |
|  | ERR2587174 | 19,810,431 | | Spleen |
|  | ERR2587177 | 12,090,753 | | Trunk kidney |
|  | ERR2587179 | 16,583,013 | | Ventricle |
|  | ERR2587181 | 14,104,635 | | White Muscle |
| *Gymnodraco acuticeps* (transcriptome) | SRR6450839 | 25,104,059 | | Pooled embryo and adult tissues |
|  | SRR6450835 | 18,230,600 | | Pooled embryo and adult tissues |
|  | SRR6450836 | 129,505,533 | | Pooled embryo tissues |
|  | SRR6450837 | 111,318,387 | | Pooled embryo tissues |
|  | SRR6450838 | 105,730,409 | | adult tissue pool (brain, gill, liver, spleen) |
| *Pseudochaenichthys georgianus* (transcriptome) † | ERR2587183 | 20,190,257 | | Blood |
|  | ERR2587177 | 12,090,753 | | Caudal Kidney |
|  | ERR2587166 | 20,526,312 | | Head kidney |
|  | ERR2587179 | 16,583,013 | | Heart (Ventricle) |
|  | ERR2587169 | 15,994,258 | | Liver |
|  | ERR2587172 | 14,510,454 | | Muscle (Pectoral) |
|  | ERR2587181 | 14,104,635 | | Muscle (White) |
|  | ERR2587171 | 11,340,950 | | Ovary |
|  | ERR2587174 | 19,810,431 | | Spleen |
| *Chaenocephalus aceratus* (transcriptome) | SRR6929344 | 21,258,801 | | Brain |
|  | SRR6929343 | 24,399,667 | | Eye |
|  | SRR6929342 | 25,536,187 | | Gill |
|  | SRR6929341 | 28,390,544 | | Heart |
|  | SRR6929348 | 25,556,533 | | Intestine |
|  | SRR6929347 | 26,931,935 | | Kidney |
|  | SRR6929346 | 39,299,440 | | Liver |
|  | SRR6929345 | 22,687,054 | | Muscle |
|  | SRR6929350 | 24,540,339 | | Ovary |
|  | SRR6929349 | 22,748,565 | | Skin |
|  | SRR6929352 | 21,889,168 | | Spleen |
|  | SRR6929351 | 28,904,859 | | Stomach |
| *Chionodraco myersi* (transcriptome) | SRR8197049 | 93,792,020 | | Liver |
|  | SRR8197051 | 106,865,971 | | Brain |
|  | SRR8197052 | 120,787,599 | | Kidney |
|  | SRR8197050 | 51,638,504 | | Spleen |
| *Chionodraco hamatus* (transcriptome) | SRR4279902 | 36,479,750 | | Gills |
|  | SRR3114175 | 28,277,980 | | Heart |
|  | SRR3114176 | 32,624,714 | | Muscle |
|  | SRR3112442 | 27,460,382 | | Liver |
|  | SRR2072638 | 17,867,714 | | N/A |
|  | SRR2072637 | 12,006,192 | | N/A |
|  | SRR2072636 | 16,830,100 | | N/A |
| Chionodraco rastrospinosus (transcriptome) | SRR5210463  (pool) | 67,052,170 | | Liver  Gill  Brain  Spleen  Heart  Head Kidney  Stomach  Intestine  Eye Cup  Lens |

Unless otherwise noted sequences were downloaded from NCBI’s SRA.

Species denoted with † were downloaded from ArrayExpress E-MTAB-6759.

Species denoted with ^x^ were downloaded from GIGADB

Species denoted with ^|^ were downloaded from NCBI’s genome assembly database

**S Table 2:**

**Genomic & Transcriptome sequences used in the data analysis (Page 1 of 2)**

| **Species** | **16S** | **ND2** | **HECW2** | **MYH6** | **PKD1** | **PPM1D** |
| --- | --- | --- | --- | --- | --- | --- |
| *Seriola dumerili* | NC_016870.1 | NC_016870.1 | ENSSDUP00000025534 ^1^ | ENSSDUT00000022504.1 ^1^ | ENSSDUT00000003940.1 ^1^ | N/A |
| *Labrus bergylta* | MT410912.1 | MT410912.1 | ENSLBEP00000029024 ^1^ | ENSLCRP00005003428 ^1^ | ENSLBET00000015305.1 ^1^ | N/A |
| *Takifugu rubripes* | AP006045.1 | AP006045.1 | ENSTRUP00000023073 ^1^ | ENSTRUP00000086471 ^1^ | N/A | N/A |
| *Larimichthys crocea* | NC_011710.1 | NC_011710.1 | ENSLCRP00005008096 ^1^ | ENSLCRP00005003428 ^1^ | ENSLCRT00005050827.1 ^1^ | N/A |
| *Gasterosteus aculeatus* | NC_041244.1 | NC_041244.1 | ENSGACP00000020039 ^1^ | ENSGACP00000018205 ^1^ | ENSGACT00000025106.1 ^1^ | N/A |
| *Cottoperca gobio* | AY520102.1 | JN186884.1 | JQ688794.1 | JN187021.1 | JQ688745.1 | N/A |
| *Eleginops maclovinus* | JN186915.1 | KF412874.1 | JQ688795.1 | JN187023.1 | JQ688746.1 | JQ688691.1 |
| *Dissostichus mawsoni* | LC138011.1 | AY256561.1 | JQ688816.1 | JN187040.1 | JQ688764.1 | JQ688715.1 |
| *Notothenia coriiceps* | NC_015653.1 | AY256563.1 | JQ688821.1 | JN187047.1 | JQ688757.1 | JQ688719.1 |
| *Pagothenia borchgrevinki* | KX025131.1 | KR153352.1 | JQ688817.1 | HM166364.1 | JQ688765.1 | † |
| *Trematomus bernacchii* | AY520126.1 | KR153464.1 | JQ688841.1 | HM166366.1 | JQ688784.1 | † |
| *Harpagifer antarcticus* | AY520130.1 | JN186894.1 | N/A | HQ169791.1 | † | JQ688717.1 |
| *Gymnodraco acuticeps* | MT559888.1 | MT559888.1 | JQ688811.1 | HQ169837.1 | JQ688759.1 | JQ688709.1 |
| *Parachaenichthys charcoti* | KP300644.1 | KP300644.1 | N/A | HQ169840.1 | † | N/A |
| *Chaenocephalus aceratus* | F933907.1 | JF933907.1 | JQ688805.1 | HQ169793.1 | † | JQ688701.1 |
| *Pseudochaenichthys georgianus* | MT559893.1 | MT559893.1 | N/A | JF264547.1 | † | N/A |
| *Chionodraco hamatus* | HQ170305.1 | HQ170101.1 | JQ688815.1 | HQ169804.1 | JQ688763.1 | † |
| *Chionodraco rastrospinosus* | AY249475.1 | AY249502.1 | N/A | HQ169808.1 | † | N/A |
| *Chionodraco myersi* | DQ526430.1 | HQ170103.1 | N/A | HQ169807.1 | † | N/A |

**S Table 2:**

**Genomic & Transcriptome sequences used in the data analysis (Page 2 of 2)**

| **Species** | **PTR** | **RHO** | **SH3PX3** | **SSRP1** | **TBR1** | **ZIC1** | **RPS71** |
| --- | --- | --- | --- | --- | --- | --- | --- |
| *Seriola dumerili* | ENSSDUT00000012076.1 ^1^ | ENSSDUT00000019767.1 ^1^ | XM_022756080.1 | ENSSDUT00000020584.1 ^1^ | XM_022740861.1 | XM_022767191.1 | N/A |
| *Labrus bergylta* | ENSLBET00000037972.1 ^1^ | ENSLBET00000012479.1 ^1^ | XM_020660907.2 | ENSLBET00000013542.1 ^1^ | XM_020644160.2 | XM_020654969.2 | N/A |
| *Takifugu rubripes* | ENSTRUT00000044496.3 ^1^ | ENSTRUT00000010811.3 ^1^ | XM_003967599.3 | ENSTRUT00000033688.3 ^1^ | XM_003961934.2 | XM_003968020.3 | N/A |
| *Larimichthys crocea* | ENSLCRT00005025024.1 ^1^ | ENSLCRT00005061767.1 ^1^ | XM_010735182.3 | ENSLCRT00005017928.1 ^1^ | XM_010732216.3 | XM_010753423.3 | N/A |
| *Gasterosteus aculeatus* | ENSGACT00000005605.1 ^1^ | EU637962.1 | XM_040162897.1 | ENSGACT00000023573.1 ^1^ | XM_040200671.1 | XM_040167334.1 | N/A |
| *Cottoperca gobio* | ENSCGOT00000017205.1 ^1^ | ENSCGOT00000016480.1 ^1^ | JN186939.1 | N/A | XM_029459088.1 | JN186803.1 | JN186844.1 |
| *Eleginops maclovinus* | HM050163.1 | AY141303.1 | JN186941.1 | N/A | JN186982.1 | JN186805.1 | JN186846.1 |
| *Dissostichus mawsoni* | JF264567.1 | DQ498794.1 | JN186958.1 | N/A | JN186999.1 | JN186822.1 | AY517753.1 |
| *Notothenia coriiceps* | HM050183.1 | XM_010795871.1 | JN186965.1 | N/A | JN187006.1 | XM_010793969.1 | FJ647673.1 |
| *Pagothenia borchgrevinki* | HM166315.1 | GU997240.1 | HM166239.1 | N/A | HM166214.1 | HM166190.1 | FJ647674.1 |
| *Trematomus bernacchii* | HM166317.1 | DQ498797.1 | HM166241.1 | N/A | HM050267.1 | HM166191.1 | FJ647676.1 |
| *Harpagifer antarcticus* | HQ169911.1 | HQ170033.1 | HQ170178.1 | N/A | HQ170237.1 | HQ169671.1 | HQ170134.1 |
| *Gymnodraco acuticeps* | HQ169958.1 | KU647485.1 | XM_034236151.1 | N/A | JF264606.1 | HQ169718.1 | HQ170165.1 |
| *Parachaenichthys charcoti* | HQ169960.1 | HQ170081.1 | HQ170226.1 | N/A | KC830426.1 | HQ169720.1 | HQ170167.1 |
| *Chaenocephalus aceratus* | HQ169914.1 | HQ170036.1 | HQ170180.1 | N/A | HQ170239.1 | HQ169674.1 | HM166091.1 |
| *Pseudochaenichthys georgianus* | HQ169943.1 | HQ170065.1 | HQ170210.1 | N/A | JF264622.1 | HQ169703.1 | HM165578.1 |
| *Chionodraco hamatus* | HQ169925.1 | HQ170047.1 | HQ170191.1 | N/A | HQ170250.1 | HQ169684.1 | HQ170142.1 |
| *Chionodraco rastrospinosus* | HQ169929.1 | HQ170050.1 | HQ170196.1 | N/A | KC830288.1 | HQ169688.1 | HM165855.1 |
| *Chionodraco myersi* | HQ169927.1 | HQ170048.1 | HQ170194.1 | N/A | HQ170049.1 | HQ169687.1 | HQ170144.1 |

^1^ denotes genes from ensemble, all other accession numbers reference NCBI’s genbank. Genes denoted with † were isolated from draft genomic annotations or transcriptomic assemblies. N/A is used to denote species gene combinations that were not included in the analysis.

**S Table 3:**

**Genes where signatures of both positive selective pressure (BUSTED or ABSREL) and relaxation of purifying selective pressure (RELAX) have been detected**

| **Comparison** | **Gene** |
| --- | --- |
|  |  |
| BUSTED & RELAX | mucolipin 3b (mcoln3b) |
| BUSTED & RELAX | microcephalin 1 (mcph1) |
| BUSTED & RELAX | kinesin family member 22 (kif22) |
|  |  |
| ABSREL & RELAX | serine/threonine-protein kinase pim-3-like |
| ABSREL & RELAX | FAST kinase domains 1 (fastkd1) |
| ABSREL & RELAX | kinetochore protein Nuf2-like |
| ABSREL & RELAX | metalloproteinase inhibitor 3 |
|  |  |
| BUSTED, ABSREL, & RELAX | apolipoprotein A-I (apoa1), mRNA |
| BUSTED, ABSREL, & RELAX | AF4/FMR2 family, member 4 (aff4) |

**S Table 4:**

**Enriched GO terms for orthogroups identified under positive selective pressure by only aBSREL in the red-blooded cryonotothenioids when compared to a set of temperate and tropical fishes**

**Biological Process (BP)**

| **GO** | **term** | **Annotated** | **Significant** | **FDR** |
| --- | --- | --- | --- | --- |
|  |  |  |  |  |
| GO:0009059 | macromolecule biosynthetic process | 2364 | 31 | 0.001102 |
| GO:0034645 | cellular macromolecule biosynthetic... | 2364 | 31 | 0.001102 |
| GO:0044249 | cellular biosynthetic process | 2365 | 31 | 0.001102 |
| GO:1901576 | organic substance biosynthetic process | 2365 | 31 | 0.001102 |
| GO:0009058 | biosynthetic process | 2367 | 31 | 0.001102 |
| GO:0044271 | cellular nitrogen compound biosynthetic ... | 2027 | 28 | 0.001102 |
| GO:0010467 | gene expression | 2198 | 28 | 0.00171 |
| GO:0044260 | cellular macromolecule metabolic process | 2592 | 31 | 0.00171 |
| GO:0043170 | macromolecule metabolic process | 5099 | 49 | 0.00171 |
| GO:0006351 | transcription, DNA-templated | 1999 | 26 | 0.00171 |
| GO:0019438 | aromatic compound biosynthetic process | 1999 | 26 | 0.00171 |
| GO:0032774 | RNA biosynthetic process | 1999 | 26 | 0.00171 |
| GO:0034654 | nucleobase-containing compound... | 1999 | 26 | 0.00171 |
| GO:0097659 | nucleic acid-templated transcription | 1999 | 26 | 0.00171 |
| GO:0018130 | heterocycle biosynthetic process | 2000 | 26 | 0.00171 |
| GO:1901362 | organic cyclic compound biosynthetic pro... | 2000 | 26 | 0.00171 |
| GO:0006355 | regulation of transcription, DNA-templat... | 1898 | 25 | 0.00171 |
| GO:1903506 | regulation of nucleic acid-templated tra... | 1898 | 25 | 0.00171 |
| GO:2001141 | regulation of RNA biosynthetic process | 1898 | 25 | 0.00171 |
| GO:0051252 | regulation of RNA metabolic process | 1899 | 25 | 0.00171 |
| GO:0019219 | regulation of nucleobase-containing... | 1900 | 25 | 0.00171 |
| GO:0010556 | regulation of macromolecule biosynthetic... | 1901 | 25 | 0.00171 |
| GO:0031326 | regulation of cellular biosynthetic proc... | 1901 | 25 | 0.00171 |
| GO:2000112 | regulation of cellular macromolecule bio... | 1901 | 25 | 0.00171 |
| GO:0009889 | regulation of biosynthetic process | 1903 | 25 | 0.00171 |
| GO:0051171 | regulation of nitrogen compound... | 1903 | 25 | 0.00171 |
| GO:0080090 | regulation of primary metabolic process | 1903 | 25 | 0.00171 |
| GO:0031323 | regulation of cellular metabolic process | 1906 | 25 | 0.00171 |
| GO:0050794 | regulation of cellular process | 1924 | 25 | 0.001966 |
| GO:0044238 | primary metabolic process | 6147 | 55 | 0.00228 |
| GO:0010468 | regulation of gene expression | 1956 | 25 | 0.002345 |
| GO:0060255 | regulation of macromolecule metabolic... | 1957 | 25 | 0.002345 |
| GO:0019222 | regulation of metabolic process | 1965 | 25 | 0.002345 |
| GO:0006807 | nitrogen compound metabolic process | 5398 | 50 | 0.002345 |
| GO:0071704 | organic substance metabolic process | 6349 | 56 | 0.002345 |
| GO:0050789 | regulation of biological process | 1986 | 25 | 0.002597 |
| GO:0016070 | RNA metabolic process | 2495 | 29 | 0.002835 |
| GO:0008152 | metabolic process | 6587 | 57 | 0.00318 |
| GO:0065007 | biological regulation | 2213 | 26 | 0.005145 |
| GO:0034641 | cellular nitrogen compound metabolic... | 3083 | 32 | 0.009006 |
| GO:0090304 | nucleic acid metabolic process | 2777 | 29 | 0.014795 |
| GO:0006139 | nucleobase-containing compound... | 3056 | 31 | 0.014795 |
| GO:0046483 | heterocycle metabolic process | 3057 | 31 | 0.014795 |
| GO:1901360 | organic cyclic compound metabolic... | 3057 | 31 | 0.014795 |
| GO:0006725 | cellular aromatic compound metabolic... | 3060 | 31 | 0.014795 |
| GO:0044237 | cellular metabolic process | 3864 | 36 | 0.024683 |
| GO:0006457 | protein folding | 62 | 3 | 0.034491 |
| GO:0002181 | cytoplasmic translation | 21 | 2 | 0.03667 |
| GO:0006412 | translation | 28 | 2 | 0.058118 |
| GO:0006518 | peptide metabolic process | 28 | 2 | 0.058118 |
| GO:0043043 | peptide biosynthetic process | 28 | 2 | 0.058118 |
| GO:0043603 | cellular amide metabolic process | 28 | 2 | 0.058118 |
| GO:0043604 | amide biosynthetic process | 28 | 2 | 0.058118 |
| GO:0044085 | cellular component biogenesis | 941 | 12 | 0.064178 |
| GO:1901564 | organonitrogen compound metabolic... | 2858 | 27 | 0.065042 |
| GO:0019538 | protein metabolic process | 2634 | 25 | 0.0798 |
| GO:0006913 | nucleocytoplasmic transport | 93 | 3 | 0.083417 |
| GO:0051169 | nuclear transport | 93 | 3 | 0.083417 |

**Molecular Function (MF)**

No significant terms were identified in this ontogeny

**Cellular Component (CC)**

| **GO** | **term** | **Annotated** | **Significant** | **FDR P-value** |
| --- | --- | --- | --- | --- |
|  |  |  |  |  |
| GO:0005622 | intracellular anatomical structure | 8075 | 71 | 0.003363 |
| GO:0043226 | organelle | 7939 | 69 | 0.00649 |
| GO:0043229 | intracellular organelle | 7152 | 64 | 0.00649 |
| GO:0043231 | intracellular membrane-bounded … | 6269 | 58 | 0.007375 |
| GO:0043227 | membrane-bounded organelle | 6636 | 60 | 0.007788 |
| GO:0005737 | cytoplasm | 4130 | 42 | 0.012488 |
| GO:0005634 | nucleus | 4328 | 42 | 0.029669 |

**S Table 5:**

**Enriched GO terms for orthogroups identified under positive selective pressure by only BUSTED in the red-blooded cryonotothenioids when compared to a set of temperate and tropical fishes**

**Biological Process (BP)**

No significant terms were identified in this ontogeny

**Molecular Function (MF)**

No significant terms were identified in this ontogeny

**Cellular Component (CC)**

No significant terms were identified in this ontogeny

**S Table 6:**

**Enriched GO terms for orthogroups identified under positive selective pressure by either (union) BUSTED or aBSREL in the red-blooded cryonotothenioids when compared to a set of temperate and tropical fishes**

**Biological Process (BP)**

| **GO** | **term** | **Annotated** | **Significant** | **FDR** |
| --- | --- | --- | --- | --- |
|  |  |  |  |  |
| GO:0044271 | cellular nitrogen compound biosynthetic ... | 407 | 32 | 0.013843 |
| GO:0006355 | regulation of transcription, DNA-templat... | 366 | 29 | 0.013843 |
| GO:1903506 | regulation of nucleic acid-templated tra... | 366 | 29 | 0.013843 |
| GO:2001141 | regulation of RNA biosynthetic process | 366 | 29 | 0.013843 |
| GO:0009889 | regulation of biosynthetic process | 367 | 29 | 0.013843 |
| GO:0010556 | regulation of macromolecule biosynthetic... | 367 | 29 | 0.013843 |
| GO:0019219 | regulation of nucleobase-containing comp... | 367 | 29 | 0.013843 |
| GO:0031326 | regulation of cellular biosynthetic proc... | 367 | 29 | 0.013843 |
| GO:0051252 | regulation of RNA metabolic process | 367 | 29 | 0.013843 |
| GO:2000112 | regulation of cellular macromolecule bio... | 367 | 29 | 0.013843 |
| GO:0031323 | regulation of cellular metabolic process | 368 | 29 | 0.013843 |
| GO:0051171 | regulation of nitrogen compound metaboli... | 368 | 29 | 0.013843 |
| GO:0080090 | regulation of primary metabolic process | 368 | 29 | 0.013843 |
| GO:0050794 | regulation of cellular process | 372 | 29 | 0.013843 |
| GO:0009058 | biosynthetic process | 483 | 35 | 0.013843 |
| GO:0009059 | macromolecule biosynthetic process | 483 | 35 | 0.013843 |
| GO:0034645 | cellular macromolecule biosynthetic proc... | 483 | 35 | 0.013843 |
| GO:0044249 | cellular biosynthetic process | 483 | 35 | 0.013843 |
| GO:1901576 | organic substance biosynthetic process | 483 | 35 | 0.013843 |
| GO:0010468 | regulation of gene expression | 378 | 29 | 0.013843 |
| GO:0060255 | regulation of macromolecule metabolic pr... | 378 | 29 | 0.013843 |
| GO:0019222 | regulation of metabolic process | 379 | 29 | 0.013843 |
| GO:0006351 | transcription, DNA-templated | 397 | 30 | 0.013843 |
| GO:0018130 | heterocycle biosynthetic process | 397 | 30 | 0.013843 |
| GO:0019438 | aromatic compound biosynthetic process | 397 | 30 | 0.013843 |
| GO:0032774 | RNA biosynthetic process | 397 | 30 | 0.013843 |
| GO:0034654 | nucleobase-containing compound... | 397 | 30 | 0.013843 |
| GO:0097659 | nucleic acid-templated transcription | 397 | 30 | 0.013843 |
| GO:1901362 | organic cyclic compound biosynthetic pro... | 397 | 30 | 0.013843 |
| GO:0050789 | regulation of biological process | 384 | 29 | 0.016432 |
| GO:0044260 | cellular macromolecule metabolic process | 516 | 36 | 0.017378 |
| GO:0010467 | gene expression | 457 | 32 | 0.030095 |
| GO:0065007 | biological regulation | 428 | 30 | 0.039268 |
| GO:0022402 | cell cycle process | 54 | 7 | 0.058424 |
| GO:0007049 | cell cycle | 131 | 12 | 0.07803 |
| GO:0007155 | cell adhesion | 88 | 9 | 0.089215 |
| GO:0022610 | biological adhesion | 88 | 9 | 0.089215 |
| GO:0000278 | mitotic cell cycle | 121 | 11 | 0.097136 |
| GO:0140014 | mitotic nuclear division | 35 | 5 | 0.097136 |
| GO:1903047 | mitotic cell cycle process | 35 | 5 | 0.097136 |

**Molecular Function (MF)**

No significant terms were identified in this ontogeny

**Cellular Component (CC)**

No significant terms were identified in this ontogeny

**S Table 7:**

**Enriched GO terms for orthogroups identified under positive selective pressure by both (intersection) BUSTED and aBSREL in the red-blooded cryonotothenioids when compared to a set of temperate and tropical fishes**

**Biological Process (BP)**

| **GO** | **term** | **Annotated** | **Significant** | **FDR** |
| --- | --- | --- | --- | --- |
|  |  |  |  |  |
| GO:0044271 | cellular nitrogen compound biosynthetic ... | 407 | 11 | 0.0984 |
| GO:0010467 | gene expression | 457 | 11 | 0.0984 |
| GO:0006351 | transcription, DNA-templated | 397 | 10 | 0.0984 |
| GO:0018130 | heterocycle biosynthetic process | 397 | 10 | 0.0984 |
| GO:0019438 | aromatic compound biosynthetic process | 397 | 10 | 0.0984 |
| GO:0032774 | RNA biosynthetic process | 397 | 10 | 0.0984 |
| GO:0034654 | nucleobase-containing compound... | 397 | 10 | 0.0984 |
| GO:0097659 | nucleic acid-templated transcription | 397 | 10 | 0.0984 |
| GO:1901362 | organic cyclic compound biosynthetic pro... | 397 | 10 | 0.0984 |
| GO:0009058 | biosynthetic process | 483 | 11 | 0.0984 |
| GO:0009059 | macromolecule biosynthetic process | 483 | 11 | 0.0984 |
| GO:0034645 | cellular macromolecule biosynthetic proc... | 483 | 11 | 0.0984 |
| GO:0044249 | cellular biosynthetic process | 483 | 11 | 0.0984 |
| GO:1901576 | organic substance biosynthetic process | 483 | 11 | 0.0984 |
| GO:0006355 | regulation of transcription, DNA-templat... | 366 | 9 | 0.0984 |
| GO:1903506 | regulation of nucleic acid-templated tra... | 366 | 9 | 0.0984 |
| GO:2001141 | regulation of RNA biosynthetic process | 366 | 9 | 0.0984 |
| GO:0009889 | regulation of biosynthetic process | 367 | 9 | 0.0984 |
| GO:0010556 | regulation of macromolecule biosynthetic... | 367 | 9 | 0.0984 |
| GO:0019219 | regulation of nucleobase-containing comp... | 367 | 9 | 0.0984 |
| GO:0031326 | regulation of cellular biosynthetic proc... | 367 | 9 | 0.0984 |
| GO:0051252 | regulation of RNA metabolic process | 367 | 9 | 0.0984 |
| GO:2000112 | regulation of cellular macromolecule bio... | 367 | 9 | 0.0984 |
| GO:0031323 | regulation of cellular metabolic process | 368 | 9 | 0.0984 |
| GO:0051171 | regulation of nitrogen compound metaboli... | 368 | 9 | 0.0984 |
| GO:0080090 | regulation of primary metabolic process | 368 | 9 | 0.0984 |
| GO:0050794 | regulation of cellular process | 372 | 9 | 0.099303 |
| GO:0044260 | cellular macromolecule metabolic process | 516 | 11 | 0.099303 |
| GO:0010468 | regulation of gene expression | 378 | 9 | 0.099303 |
| GO:0060255 | regulation of macromolecule metabolic pr... | 378 | 9 | 0.099303 |
| GO:0019222 | regulation of metabolic process | 379 | 9 | 0.099303 |

**Molecular Function (MF)**

No significant terms were identified in this ontogeny

**Cellular Component (CC)**

No significant terms were identified in this ontogeny

**S Table 8:**

**The 42 genes identified under positive selection by both (intersection) BUSTED and aBSREL in the red-blooded cryonotothenioids**

| ***D. mawsoni* contig ID** | **Accession** | **Gene Description** |
| --- | --- | --- |
|  |  |  |
|  |  |  |
| Dissostichus_mawsoni_GLEAN_10004726 | XM_034134301.1 | actin-related protein 3-like |
| Dissostichus_mawsoni_GLEAN_10014834 | XM_034081152.1 | abhydrolase domain containing 17A, depalmitoylase b (abhd17ab) |
| Dissostichus_mawsoni_GLEAN_10020511 | XM_034151765.1 | membrane-associated ring finger (C3HC4) 8 (march8) |
| Dissostichus_mawsoni_GLEAN_10000924 | XM_034208437.1 | fatty acid-binding protein, brain |
| Dissostichus_mawsoni_GLEAN_10005717 | XM_034125884.1 | chondroitin sulfate synthase 3-like |
| Dissostichus_mawsoni_GLEAN_10020328 | XM_034100676.1 | leucine-rich pentatricopeptide repeat containing (lrpprc) |
| Dissostichus_mawsoni_GLEAN_10020700 | XM_010793323.1 | PR domain containing 4 (prdm4) |
| Dissostichus_mawsoni_GLEAN_10004964 | XM_034141688.1 | alanyl-tRNA synthetase 1 (aars1) |
| Dissostichus_mawsoni_GLEAN_10010870 | XM_034120672.1 | programmed cell death 4b (pdcd4b) |
| Dissostichus_mawsoni_GLEAN_10011890 | XM_034139887.1 | AF4/FMR2 family, member 4 (aff4) |
| Dissostichus_mawsoni_GLEAN_10018274 | XM_034119651.1 | interferon regulatory factor 3 (irf3) |
| Dissostichus_mawsoni_GLEAN_10017027 | XM_034117028.1 | 5-aminoimidazole-4-carboxamide ribonucleotide formyltransferase/IMP cyclohydrolase (atic) |
| Dissostichus_mawsoni_GLEAN_10022233 | XM_034139949.1 | aldolase C, fructose-bisphosphate, b (aldocb) |
| Dissostichus_mawsoni_GLEAN_10005573 | XM_034113336.1 | upstream stimulatory factor 1-like |
| Dissostichus_mawsoni_GLEAN_10011174 | XM_034127844.1 | eukaryotic translation initiation factor 3 subunit H |
| Dissostichus_mawsoni_GLEAN_10016863 | XM_034122416.1 | HO complex 5 (thoc5) |
| Dissostichus_mawsoni_GLEAN_10016354 | XM_034080971.1 | DCN1, defective in cullin neddylation 1, domain containing 4 |
| Dissostichus_mawsoni_GLEAN_10013849 | XM_034209209.1 | DDB1 and CUL4 associated factor 8 (dcaf8) |
| Dissostichus_mawsoni_GLEAN_10016301 | XM_034229776.1 | phospholipid phosphatase 3 (plpp3) |
| Dissostichus_mawsoni_GLEAN_10008024 | XM_034219370.1 | BRCA1 associated protein-1 (ubiquitin carboxy-terminal hydrolase) (bap1) |
| Dissostichus_mawsoni_GLEAN_10020049 | XM_034125057.1 | CAS1 domain containing 1 (casd1) |
| Dissostichus_mawsoni_GLEAN_10019276 | XM_034141431.1 | dual specificity phosphatase 8a (dusp8a) |
| Dissostichus_mawsoni_GLEAN_10019851 | XM_034125317.1 | proline rich mitotic checkpoint control factor (prcc) |
| Dissostichus_mawsoni_GLEAN_10013954 | XM_034235156.1 | neutral cholesterol ester hydrolase 1a (nceh1a) |
| Dissostichus_mawsoni_GLEAN_10016303 | XM_034229927.1 | biogenesis of lysosomal organelles complex-1, subunit 2 (bloc1s2) |
| Dissostichus_mawsoni_GLEAN_10017720 | XM_034128873.1 | immunoglobulin-like domain containing receptor 1b (ildr1b) |
| Dissostichus_mawsoni_GLEAN_10018727 | XM_034205847.1 | zinc finger DHHC-type palmitoyltransferase 23b (zdhhc23b) |
| Dissostichus_mawsoni_GLEAN_10019763 | XM_010793878.1 | apolipoprotein A-I (apoa1) |
| Dissostichus_mawsoni_GLEAN_10002615 | XM_034112977.1 | WD repeat and SOCS box containing 1 (wsb1) |
| Dissostichus_mawsoni_GLEAN_10012048 | XM_034119462.1 | family with sequence similarity 53 member B (fam53b) |
| Dissostichus_mawsoni_GLEAN_10018841 | XM_010771106.1 | MAF1 homolog (S. cerevisiae) (maf1) |
| Dissostichus_mawsoni_GLEAN_10020323 | XM_034238009.1 | histidine triad nucleotide binding protein 1 (hint1) |
| Dissostichus_mawsoni_GLEAN_10008420 | XM_034088333.1 | Jupiter microtubule associated homolog 2 (jpt2) |
| Dissostichus_mawsoni_GLEAN_10010015 | XM_034143427.1 | phosphatidylcholine transfer protein (pctp) |
| Dissostichus_mawsoni_GLEAN_10012759 | XM_034224093.1 | mitochondrial ribosomal protein S24 (mrps24) |
| Dissostichus_mawsoni_GLEAN_10008527 | XM_034121986.1 | CDC42 effector protein (Rho GTPase binding) 3 (cdc42ep3) |
| Dissostichus_mawsoni_GLEAN_10005600 | XM_034145192.1 | zinc finger, CCCH-type with G patch domain (zgpat) |
| Dissostichus_mawsoni_GLEAN_10003919 | XM_034078357.1 | N-acetyltransferase 15 (GCN5-related, putative) (nat15) |
| Dissostichus_mawsoni_GLEAN_10010080 | XM_034127238.1 | phosphatidylinositol glycan anchor biosynthesis, class V (pigv) |
| Dissostichus_mawsoni_GLEAN_10010308 | XM_010776739.1 | transcription factor B1, mitochondrial (tfb1m) |
| Dissostichus_mawsoni_GLEAN_10012407 | XM_034220284.1 | insulin-like growth factor binding protein, acid labile subunit (igfals) |
| Dissostichus_mawsoni_GLEAN_10021202 | XM_034137357.1 | IFI30 lysosomal thiol reductase (ifi30) |

The *D. mawsoni* contig ID column shows the contig ID for the *D. mawsoni* member of each significant orthogroup based on the predicted peptide naming used in assembly from Chen et al. 2019

The Accession column shows the accession number of the best blast hit from searching NCBI’s nt database for that gene

The gene description column shows the descriptive name for the orthogroup based on the BLAST search against NCBI’s nt database

**S Table 9:**

**The 160 genes identified by either (union) BUSTED or aBSREL as experiencing positive selection in the red-blooded cryonotothenioids**

| ***D. mawsoni* contig ID** | **Accession** | **Gene Description** |
| --- | --- | --- |
|  |  |  |
| Dissostichus_mawsoni_GLEAN_10000321 | XM_034130967.1 | regulatory factor X, 5 (rfx5) |
| Dissostichus_mawsoni_GLEAN_10000924 | XM_034208437.1 | fatty acid-binding protein, brain |
| Dissostichus_mawsoni_GLEAN_10001174 | XM_034221878.1 | signal transducer and activator of transcription 1a (stat1a) |
| Dissostichus_mawsoni_GLEAN_10001356 | XM_034223238.1 | B-cadherin-like |
| Dissostichus_mawsoni_GLEAN_10001377 | XM_034126967.1 | nuclear factor of activated T-cells, cytoplasmic 3-like |
| Dissostichus_mawsoni_GLEAN_10001507 | XM_034135543.1 | metalloproteinase inhibitor 3 |
| Dissostichus_mawsoni_GLEAN_10001519 | XM_034222799.1 | long-chain-fatty-acid--CoA ligase 1-like |
| Dissostichus_mawsoni_GLEAN_10001652 | XM_034127118.1 | VPS35 retromer complex component (vps35) |
| Dissostichus_mawsoni_GLEAN_10001714 | XM_034232872.1 | K(lysine) acetyltransferase 8 (kat8) |
| Dissostichus_mawsoni_GLEAN_10001716 | XM_034220322.1 | GRB2 related adaptor protein 2a (grap2a) |
| Dissostichus_mawsoni_GLEAN_10002470 | XM_034143163.1 | sine oculis-binding protein homolog B-like |
| Dissostichus_mawsoni_GLEAN_10002477 | XM_034120925.1 | teneurin-3 |
| Dissostichus_mawsoni_GLEAN_10002615 | XM_034112977.1 | WD repeat and SOCS box containing 1 (wsb1) |
| Dissostichus_mawsoni_GLEAN_10003096 | XM_034136871.1 | solute carrier family 7 member 7 (slc7a7) |
| Dissostichus_mawsoni_GLEAN_10003175 | XM_010766631.1 | cyclin T2 (ccnt2) |
| Dissostichus_mawsoni_GLEAN_10003197 | XM_034202218.1 | N-6 adenine-specific DNA methyltransferase 1 (n6amt1) |
| Dissostichus_mawsoni_GLEAN_10003224 | XM_034144679.1 | FKBP prolyl isomerase 1Ab (fkbp1ab) |
| Dissostichus_mawsoni_GLEAN_10003726 | XM_034225238.1 | long-chain-fatty-acid--CoA ligase ACSBG2-like |
| Dissostichus_mawsoni_GLEAN_10003919 | XM_034078357.1 | N-acetyltransferase 15 (GCN5-related, putative) (nat15) |
| Dissostichus_mawsoni_GLEAN_10003984 | XM_034201209.1 | von Hippel-Lindau binding protein 1 (vbp1) |
| Dissostichus_mawsoni_GLEAN_10004584 | XM_034079902.1 | dual specificity protein phosphatase CDC14AB-like |
| Dissostichus_mawsoni_GLEAN_10004631 | XM_034235770.1 | FERM, ARHGEF and pleckstrin domain-containing protein 1-like |
| Dissostichus_mawsoni_GLEAN_10004689 | XM_034123436.1 | transmembrane protein 120B (tmem120b) |
| Dissostichus_mawsoni_GLEAN_10004700 | XM_034121512.1 | UF2 component of NDC80 kinetochore complex (nuf2) |
| Dissostichus_mawsoni_GLEAN_10004726 | XM_034134301.1 | actin-related protein 3-like |
| Dissostichus_mawsoni_GLEAN_10004964 | XM_034141688.1 | alanyl-tRNA synthetase 1 (aars1) |
| Dissostichus_mawsoni_GLEAN_10005231 | EU488726.1 | immunoglobulin M heavy chain secreted form |
| Dissostichus_mawsoni_GLEAN_10005497 | XM_034216699.1 | fructose-1,6-bisphosphatase 1-like |
| Dissostichus_mawsoni_GLEAN_10005573 | XM_034113336.1 | upstream stimulatory factor 1-like |
| Dissostichus_mawsoni_GLEAN_10005578 | XM_010780413.1 | hydroxymethylbilane synthase (hmbs) |
| Dissostichus_mawsoni_GLEAN_10005600 | XM_034145192.1 | zinc finger, CCCH-type with G patch domain (zgpat) |
| Dissostichus_mawsoni_GLEAN_10005625 | XM_034120417.1 | regulatory factor X, 1b (influences HLA class II expression) (rfx1b) |
| Dissostichus_mawsoni_GLEAN_10005643 | XM_034120811.1 | pyrroline-5-carboxylate reductase 1b (pycr1b) |
| Dissostichus_mawsoni_GLEAN_10005717 | XM_034125884.1 | chondroitin sulfate synthase 3-like |
| Dissostichus_mawsoni_GLEAN_10005876 | XM_034205303.1 | dehydrogenase/reductase (SDR family) member 4 (dhrs4) |
| Dissostichus_mawsoni_GLEAN_10006966 | XM_034233695.1 | CD9 molecule a (cd9a) |
| Dissostichus_mawsoni_GLEAN_10007266 | XM_034126782.1 | serine/threonine-protein kinase pim-3-like |
| Dissostichus_mawsoni_GLEAN_10007563 | XM_034130311.1 | insulin-like growth factor-binding protein 4 |
| Dissostichus_mawsoni_GLEAN_10007881 | XM_010787488.1 | coxsackievirus and adenovirus receptor homolog |
| Dissostichus_mawsoni_GLEAN_10007894 | XM_034138651.1 | E74-like ETS transcription factor 1 (elf1) |
| Dissostichus_mawsoni_GLEAN_10007976 | XM_034100871.1 | epithelial cell adhesion molecule (epcam) |
| Dissostichus_mawsoni_GLEAN_10008024 | XM_034219370.1 | BRCA1 associated protein-1 (ubiquitin carboxy-terminal hydrolase) (bap1) |
| Dissostichus_mawsoni_GLEAN_10008244 | XM_034130829.1 | tRNA methyltransferase 10A (trmt10a) |
| Dissostichus_mawsoni_GLEAN_10008420 | XM_034088333.1 | Jupiter microtubule associated homolog 2 (jpt2) |
| Dissostichus_mawsoni_GLEAN_10008527 | XM_034121986.1 | CDC42 effector protein (Rho GTPase binding) 3 (cdc42ep3) |
| Dissostichus_mawsoni_GLEAN_10008599 | XM_034140976.1 | alpha-1,3-mannosyl-glycoprotein 4-beta-N-acetylglucosaminyltransferase C-like |
| Dissostichus_mawsoni_GLEAN_10008712 | XM_034133615.1 | ribosomal protein S16 (rps16) |
| Dissostichus_mawsoni_GLEAN_10008874 | XM_034221520.1 | PTB domain-containing engulfment adapter protein 1 |
| Dissostichus_mawsoni_GLEAN_10009305 | XM_034132100.1 | Krueppel-like factor 6 |
| Dissostichus_mawsoni_GLEAN_10009575 | XM_034233436.1 | lysozyme C-like |
| Dissostichus_mawsoni_GLEAN_10009815 | XR_004568178.1 | NIMA related kinase 3 (nek3) |
| Dissostichus_mawsoni_GLEAN_10009914 | XM_034150424.1 | mediator complex subunit 23 (med23) |
| Dissostichus_mawsoni_GLEAN_10010015 | XM_034143427.1 | phosphatidylcholine transfer protein (pctp) |
| Dissostichus_mawsoni_GLEAN_10010063 | XM_034102837.1 | mitochondrial ribosomal protein L13 (mrpl13) |
| Dissostichus_mawsoni_GLEAN_10010080 | XM_034127238.1 | phosphatidylinositol glycan anchor biosynthesis, class V (pigv) |
| Dissostichus_mawsoni_GLEAN_10010082 | XM_034127696.1 | transcription elongation factor A (SII), 3 (tcea3) |
| Dissostichus_mawsoni_GLEAN_10010306 | XM_034148196.1 | cell cycle control protein 50A-like |
| Dissostichus_mawsoni_GLEAN_10010308 | XM_010776739.1 | transcription factor B1, mitochondrial (tfb1m) |
| Dissostichus_mawsoni_GLEAN_10010316 | XM_034127456.1 | forkhead box A2 (foxa2) |
| Dissostichus_mawsoni_GLEAN_10010332 | XM_034122542.1 | LCK proto-oncogene, Src family tyrosine kinase (lck) |
| Dissostichus_mawsoni_GLEAN_10010459 | XM_034136713.1 | polyamine-modulated factor 1 |
| Dissostichus_mawsoni_GLEAN_10010507 | XM_034136574.1 | UBX domain protein 1 (ubxn1) |
| Dissostichus_mawsoni_GLEAN_10010728 | XM_034090778.1 | UTP15 small subunit processome component (utp15) |
| Dissostichus_mawsoni_GLEAN_10010743 | XM_034090829.1 | inhibitor of nuclear factor kappa B kinase subunit beta (ikbkb) |
| Dissostichus_mawsoni_GLEAN_10010870 | XM_034120672.1 | programmed cell death 4b (pdcd4b) |
| Dissostichus_mawsoni_GLEAN_10011101 | XM_034153456.1 | guanine nucleotide binding protein-like 2 (nucleolar) (gnl2) |
| Dissostichus_mawsoni_GLEAN_10011174 | XM_034127844.1 | eukaryotic translation initiation factor 3 subunit H |
| Dissostichus_mawsoni_GLEAN_10011208 | XM_010774661.1 | NADH dehydrogenase (ubiquinone) 1 beta subcomplex, 4, 15kDa (ndufb4) |
| Dissostichus_mawsoni_GLEAN_10011300 | XM_034089247.1 | desumoylating isopeptidase 1a (desi1a) |
| Dissostichus_mawsoni_GLEAN_10011324 | XM_034144134.1 | 3-mercaptopyruvate sulfurtransferase-like |
| Dissostichus_mawsoni_GLEAN_10011662 | XM_034140837.1 | eukaryotic translation initiation factor 4ea (eif4ea) |
| Dissostichus_mawsoni_GLEAN_10011890 | XM_034139887.1 | AF4/FMR2 family, member 4 (aff4) |
| Dissostichus_mawsoni_GLEAN_10012048 | XM_034119462.1 | family with sequence similarity 53 member B (fam53b) |
| Dissostichus_mawsoni_GLEAN_10012119 | XM_034211221.1 | aldehyde dehydrogenase 18 family, member A1 (aldh18a1) |
| Dissostichus_mawsoni_GLEAN_10012257 | XM_034147691.1 | N-acetyllactosaminide beta-1,3-N-acetylglucosaminyltransferase 3-like |
| Dissostichus_mawsoni_GLEAN_10012298 | XM_034081070.1 | profilin-2-like |
| Dissostichus_mawsoni_GLEAN_10012407 | XM_034220284.1 | insulin-like growth factor binding protein, acid labile subunit (igfals) |
| Dissostichus_mawsoni_GLEAN_10012524 | XM_034136872.1 | OCRL inositol polyphosphate-5-phosphatase (ocrl) |
| Dissostichus_mawsoni_GLEAN_10012734 | XM_034123529.1 | SDA1 domain containing 1 (sdad1) |
| Dissostichus_mawsoni_GLEAN_10012759 | XM_034224093.1 | mitochondrial ribosomal protein S24 (mrps24) |
| Dissostichus_mawsoni_GLEAN_10012794 | XM_034123517.1 | strawberry notch homolog 1 (Drosophila) (sbno1) |
| Dissostichus_mawsoni_GLEAN_10013275 | XM_034216044.1 | UDP-Gal:betaGlcNAc beta 1,4- galactosyltransferase, polypeptide 3 (b4galt3) |
| Dissostichus_mawsoni_GLEAN_10013325 | XM_034238900.1 | torsin family 1 (tor1) |
| Dissostichus_mawsoni_GLEAN_10013350 | XM_034123498.1 | neurolysin (metallopeptidase M3 family) (nln) |
| Dissostichus_mawsoni_GLEAN_10013405 | XM_034109047.1 | male germ cell-associated kinase (mak) |
| Dissostichus_mawsoni_GLEAN_10013417 | XM_034211929.1 | programmed cell death 6 (pdcd6) |
| Dissostichus_mawsoni_GLEAN_10013547 | XM_034150515.1 | NF-kappa-B inhibitor alpha-like |
| Dissostichus_mawsoni_GLEAN_10013849 | XM_034209209.1 | DDB1 and CUL4 associated factor 8 (dcaf8) |
| Dissostichus_mawsoni_GLEAN_10013954 | XM_034235156.1 | neutral cholesterol ester hydrolase 1a (nceh1a) |
| Dissostichus_mawsoni_GLEAN_10014068 | XM_034153377.1 | transient receptor potential cation channel, subfamily V, member 1 (trpv1), |
| Dissostichus_mawsoni_GLEAN_10014435 | XM_034140201.1 | xosome component 8 (exosc8) |
| Dissostichus_mawsoni_GLEAN_10014488 | XM_034199592.1 | transmembrane protein 47-like |
| Dissostichus_mawsoni_GLEAN_10014632 | XM_034143314.1 | RUS family member 1 (rusf1) |
| Dissostichus_mawsoni_GLEAN_10014661 | XM_034088310.1 | upstream binding transcription factor (ubtf) |
| Dissostichus_mawsoni_GLEAN_10014834 | XM_034081152.1 | abhydrolase domain containing 17A, depalmitoylase b (abhd17ab) |
| Dissostichus_mawsoni_GLEAN_10014980 | XM_034197715.1 | EFR3 homolog B |
| Dissostichus_mawsoni_GLEAN_10015284 | XM_034148378.1 | iroquois homeobox 3a (irx3a) |
| Dissostichus_mawsoni_GLEAN_10015337 | XM_034116088.1 | guanine nucleotide-binding protein subunit beta-5b-like |
| Dissostichus_mawsoni_GLEAN_10015518 | XM_034213481.1 | glycoprotein M6Bb (gpm6bb) |
| Dissostichus_mawsoni_GLEAN_10015766 | XM_034126353.1 | calcium-responsive transactivator-like |
| Dissostichus_mawsoni_GLEAN_10016197 | XM_034133414.1 | guanine nucleotide binding protein (G protein), alpha inhibiting activity polypeptide 1 (gnai1) |
| Dissostichus_mawsoni_GLEAN_10016301 | XM_034229776.1 | phospholipid phosphatase 3 (plpp3) |
| Dissostichus_mawsoni_GLEAN_10016303 | XM_034229927.1 | biogenesis of lysosomal organelles complex-1, subunit 2 (bloc1s2) |
| Dissostichus_mawsoni_GLEAN_10016354 | XM_034080971.1 | DCN1, defective in cullin neddylation 1, domain containing 4 (S. cerevisiae) (dcun1d4) |
| Dissostichus_mawsoni_GLEAN_10016373 | XM_010770717.1 | NADH dehydrogenase (ubiquinone) 1 beta subcomplex, 5, 16kDa (ndufb5) |
| Dissostichus_mawsoni_GLEAN_10016466 | XM_034236697.1 | ubiquitin-conjugating enzyme E2 E2-like |
| Dissostichus_mawsoni_GLEAN_10016495 | XM_034124703.1 | plasma kallikrein-like |
| Dissostichus_mawsoni_GLEAN_10016863 | XM_034122416.1 | THO complex 5 (thoc5) |
| Dissostichus_mawsoni_GLEAN_10017027 | XM_034117028.1 | 5-aminoimidazole-4-carboxamide ribonucleotide formyltransferase/IMP cyclohydrolase (atic) |
| Dissostichus_mawsoni_GLEAN_10017101 | XM_034116933.1 | solute carrier family 39 member 1 (slc39a1) |
| Dissostichus_mawsoni_GLEAN_10017445 | XM_034147133.1 | mucolipin 3b (mcoln3b) |
| Dissostichus_mawsoni_GLEAN_10017540 | XM_034203005.1 | homer scaffold protein 3b (homer3b) |
| Dissostichus_mawsoni_GLEAN_10017600 | XM_034213388.1 | FAST kinase domains 1 (fastkd1) |
| Dissostichus_mawsoni_GLEAN_10017720 | XM_034128873.1 | immunoglobulin-like domain containing receptor 1b (ildr1b) |
| Dissostichus_mawsoni_GLEAN_10017874 | XM_034239608.1 | isocitrate dehydrogenase (NAD(+)) 3 non-catalytic subunit beta (idh3b) |
| Dissostichus_mawsoni_GLEAN_10017965 | XM_034123132.1 | B-box and SPRY domain containing (bspry) |
| Dissostichus_mawsoni_GLEAN_10018029 | XM_034123455.1 | developmentally regulated GTP binding protein 1 (drg1) |
| Dissostichus_mawsoni_GLEAN_10018172 | XM_034119740.1 | angiotensin I converting enzyme (peptidyl-dipeptidase A) 1 (ace) |
| Dissostichus_mawsoni_GLEAN_10018274 | XM_034119651.1 | interferon regulatory factor 3 (irf3) |
| Dissostichus_mawsoni_GLEAN_10018296 | XM_034119457.1 | kinesin family member 22 (kif22) |
| Dissostichus_mawsoni_GLEAN_10018348 | XM_034120083.1 | attractin-like 1b (atrnl1b) |
| Dissostichus_mawsoni_GLEAN_10018459 | XM_034134628.1 | CASP8 and FADD-like apoptosis regulator |
| Dissostichus_mawsoni_GLEAN_10018572 | XM_034236108.1 | peptidylprolyl isomerase B (cyclophilin B) (ppib) |
| Dissostichus_mawsoni_GLEAN_10018578 | XM_034149249.1 | iron-responsive element binding protein 2 (ireb2) |
| Dissostichus_mawsoni_GLEAN_10018727 | XM_034205847.1 | zinc finger DHHC-type palmitoyltransferase 23b (zdhhc23b) |
| Dissostichus_mawsoni_GLEAN_10018754 | XM_034229648.1 | armadillo repeat containing 1 (armc1) |
| Dissostichus_mawsoni_GLEAN_10018841 | XM_010771106.1 | MAF1 homolog (S. cerevisiae) (maf1) |
| Dissostichus_mawsoni_GLEAN_10018954 | XM_010787156.1 | ectodysplasin A (eda) |
| Dissostichus_mawsoni_GLEAN_10018996 | XM_034121472.1 | catenin (cadherin-associated protein), alpha 1 (ctnna1) |
| Dissostichus_mawsoni_GLEAN_10019121 | XM_034198939.1 | neuregulin 2b (nrg2b) |
| Dissostichus_mawsoni_GLEAN_10019276 | XM_034141431.1 | dual specificity phosphatase 8a (dusp8a) |
| Dissostichus_mawsoni_GLEAN_10019398 | XM_034138794.1 | replication termination factor 2 (rtf2) |
| Dissostichus_mawsoni_GLEAN_10019405 | XM_034198449.1 | TBC1 domain family, member 20 (tbc1d20) |
| Dissostichus_mawsoni_GLEAN_10019524 | XM_034223802.1 | activating transcription factor 1 (atf1) |
| Dissostichus_mawsoni_GLEAN_10019636 | XM_034115268.1 | phosphodiesterase 9A like (pde9al) |
| Dissostichus_mawsoni_GLEAN_10019688 | XM_034115066.1 | uncharacterized protein |
| Dissostichus_mawsoni_GLEAN_10019734 | XM_034233207.1 | P450 (cytochrome) oxidoreductase b (porb) |
| Dissostichus_mawsoni_GLEAN_10019763 | XM_010793878.1 | apolipoprotein A-I (apoa1) |
| Dissostichus_mawsoni_GLEAN_10019851 | XM_034125317.1 | proline rich mitotic checkpoint control factor (prcc) |
| Dissostichus_mawsoni_GLEAN_10019884 | XM_034125248.1 | antifreeze protein type IV (afp4) |
| Dissostichus_mawsoni_GLEAN_10019981 | XM_034240938.1 | programmed cell death 6 interacting protein (pdcd6ip) |
| Dissostichus_mawsoni_GLEAN_10020049 | XM_034125057.1 | CAS1 domain containing 1 (casd1) |
| Dissostichus_mawsoni_GLEAN_10020231 | XM_034118378.1 | macrophage-expressed gene 1 protein-like |
| Dissostichus_mawsoni_GLEAN_10020323 | XM_034238009.1 | histidine triad nucleotide binding protein 1 (hint1) |
| Dissostichus_mawsoni_GLEAN_10020328 | XM_034100676.1 | leucine-rich pentatricopeptide repeat containing (lrpprc) |
| Dissostichus_mawsoni_GLEAN_10020463 | XM_034152071.1 | inorganic pyrophosphatase 1b (ppa1b) |
| Dissostichus_mawsoni_GLEAN_10020511 | XM_034151765.1 | membrane-associated ring finger (C3HC4) 8 (march8) |
| Dissostichus_mawsoni_GLEAN_10020585 | XM_034100253.1 | microcephalin 1 (mcph1) |
| Dissostichus_mawsoni_GLEAN_10020593 | XM_034151865.1 | glutamate-cysteine ligase, catalytic subunit (gclc) |
| Dissostichus_mawsoni_GLEAN_10020700 | XM_010793323.1 | PR domain containing 4 (prdm4) |
| Dissostichus_mawsoni_GLEAN_10020877 | XM_010791440.1 | transcription elongation factor A (SII), 1 (tcea1) |
| Dissostichus_mawsoni_GLEAN_10021003 | XM_034137302.1 | desmocollin 2 like (dsc2l) |
| Dissostichus_mawsoni_GLEAN_10021202 | XM_034137357.1 | IFI30 lysosomal thiol reductase (ifi30) |
| Dissostichus_mawsoni_GLEAN_10021719 | XM_034231472.1 | endothelin receptor type Aa (ednraa) |
| Dissostichus_mawsoni_GLEAN_10021747 | XM_034231541.1 | mediator complex subunit 28 (med28) |
| Dissostichus_mawsoni_GLEAN_10022152 | XM_034240554.1 | F11 receptor, tandem duplicate 1 (f11r.1) |
| Dissostichus_mawsoni_GLEAN_10022222 | XM_034201657.1 | inositol polyphosphate-5-phosphatase Kb (inpp5kb) |
| Dissostichus_mawsoni_GLEAN_10022233 | XM_034139949.1 | aldolase C, fructose-bisphosphate, b (aldocb) |
| Dissostichus_mawsoni_GLEAN_10022416 | XM_034116039.1 | guanine nucleotide binding protein (G protein), alpha activating activity polypeptide O, b (gnao1b) |
| Dissostichus_mawsoni_GLEAN_10022436 | XM_034116358.1 | RNA polymerase II subunit M (polr2m) |

The *D. mawsoni* contig ID column shows the contig ID for the *D. mawsoni* member of each significant orthogroup based on the predicted peptide naming used in assembly from Chen et al. 2019

The Accession column shows the accession number of the best blast hit from searching NCBI’s nt database for that gene

The gene description column shows the descriptive name for the orthogroup based on the BLAST search against NCBI’s nt database

**S Table 10:**

**Enriched GO terms for orthogroups consistently identified under relaxed selective pressure by RELAX in the red-blooded cryonotothenioids when compared to a set of temperate and tropical fishes**

**Biological Process (BP)**

No significant terms were identified in this ontogeny

**Molecular Function (MF)**

No significant terms were identified in this ontogeny

**Cellular Component (CC)**

| **GO** | **term** | **Annotated** | **Significant** | **FDR** |
| --- | --- | --- | --- | --- |
|  |  |  |  |  |
| GO:0005739 | mitochondrion | 325 | 23 | 0.00912 |

**S Table 11:**

**The genes identified by RELAX as experiencing a significant relaxation of selective pressure in the red-blooded cryonotothenioids**

| ***D. mawsoni* contig ID** | **K** | **FDR** | **Accession** | **Gene Description** |
| --- | --- | --- | --- | --- |
|  |  |  |  |  |
| Dissostichus_mawsoni_GLEAN_10021925 | 0.041399 | 7.33E-11 | XM_034082808.1 | transcription elongation factor, mitochondrial (tefm) |
| Dissostichus_mawsoni_GLEAN_10017600 | 0 | 8.31E-11 | XM_034213388.1 | FAST kinase domains 1 (fastkd1) |
| Dissostichus_mawsoni_GLEAN_10001548 | 0.2009 | 2.70E-09 | XM_034150329.1 | major facilitator superfamily domain containing 4B (mfsd4b) |
| Dissostichus_mawsoni_GLEAN_10017828 | 0 | 1.22E-07 | XM_034212337.1 | cell death activator CIDE-3-like |
| Dissostichus_mawsoni_GLEAN_10012052 | 0.398755 | 1.34E-06 | XM_034119929.1 | carbohydrate sulfotransferase 15-like |
| Dissostichus_mawsoni_GLEAN_10018296 | 0.406378 | 2.94E-06 | XM_034119457.1 | kinesin family member 22 (kif22) |
| Dissostichus_mawsoni_GLEAN_10019762 | 0.283827 | 5.57E-06 | XM_034202803.1 | beta-secretase 1 (bace1) |
| Dissostichus_mawsoni_GLEAN_10021275 | 0.347649 | 2.17E-05 | XM_034226245.1 | yippee-like 5 |
| Dissostichus_mawsoni_GLEAN_10019040 | 0.367808 | 0.000158 | XM_034121160.1 | cytoplasmic FMR1 interacting protein 2 (cyfip2) |
| Dissostichus_mawsoni_GLEAN_10013160 | 0.120065 | 0.000165 | XM_034144922.1 | signal transducer and activator of transcription 6, interleukin-4 induced (stat6) |
| Dissostichus_mawsoni_GLEAN_10002221 | 0.236628 | 0.000186 | XM_034205048.1 | TBC1 domain family, member 22B (tbc1d22b) |
| Dissostichus_mawsoni_GLEAN_10000642 | 0.345605 | 0.000186 | XM_034133156.1 | spindlin-Z-like |
| Dissostichus_mawsoni_GLEAN_10018859 | 4.82E-05 | 0.000243 | XM_034214072.1 | FAST kinase domains 3 (fastkd3) |
| Dissostichus_mawsoni_GLEAN_10018318 | 0.006327 | 0.000327 | XM_034120175.1 | translational activator of cytochrome c oxidase 1-like |
| Dissostichus_mawsoni_GLEAN_10011176 | 0.646979 | 0.000536 | XM_034101906.1 | exostosin-1a-like |
| Dissostichus_mawsoni_GLEAN_10020607 | 0.508629 | 0.000536 | XM_034131534.1 | proteasome 26S subunit, ATPase 2 (psmc2) |
| Dissostichus_mawsoni_GLEAN_10020404 | 0.199428 | 0.000536 | XM_034152439.1 | carbohydrate sulfotransferase 15-like |
| Dissostichus_mawsoni_GLEAN_10004700 | 0.021001 | 0.000536 | XM_034121512.1 | UF2 component of NDC80 kinetochore complex (nuf2) |
| Dissostichus_mawsoni_GLEAN_10017141 | 0.35854 | 0.000816 | XM_034116612.1 | solute carrier family 25 member 44-like |
| Dissostichus_mawsoni_GLEAN_10003577 | 0.092871 | 0.001048 | XM_044198970.1 | receptor accessory protein 6 (reep6) |
| Dissostichus_mawsoni_GLEAN_10007266 | 0.016361 | 0.001286 | XM_034126782.1 | serine/threonine-protein kinase pim-3-like |
| Dissostichus_mawsoni_GLEAN_10020585 | 0 | 0.002154 | XM_034100253.1 | microcephalin 1 (mcph1) |
| Dissostichus_mawsoni_GLEAN_10007940 | 0.39531 | 0.002368 | XM_034225706.1 | sphingosine-1-phosphate lyase 1 (sgpl1) |
| Dissostichus_mawsoni_GLEAN_10018042 | 0.505179 | 0.002368 | XM_034122828.1 | nicotinamide nucleotide transhydrogenase (nnt) |
| Dissostichus_mawsoni_GLEAN_10012998 | 0.057166 | 0.002368 | XM_034127020.1 | transmembrane protein 205 (tmem205) |
| Dissostichus_mawsoni_GLEAN_10020189 | 0.416238 | 0.002603 | XM_034118276.1 | meningioma 1b (mn1b) |
| Dissostichus_mawsoni_GLEAN_10019425 | 0.198491 | 0.002761 | XM_010776843.1 | family with sequence similarity 210, member B (fam210b) |
| Dissostichus_mawsoni_GLEAN_10021166 | 0.602669 | 0.002893 | XM_034138033.1 | fem-1 homolog a (fem1a) |
| Dissostichus_mawsoni_GLEAN_10022472 | 0.360273 | 0.003079 | XM_034116280.1 | TEA domain family member 1b (tead1b) |
| Dissostichus_mawsoni_GLEAN_10016268 | 0.095813 | 0.003181 | XM_034081738.1 | cytochrome c oxidase assembly factor 7 |
| Dissostichus_mawsoni_GLEAN_10006481 | 0.220343 | 0.003389 | XM_034138712.1 | solute carrier family 2 member 1b (slc2a1b) |
| Dissostichus_mawsoni_GLEAN_10018048 | 0 | 0.004061 | XM_034096519.1 | NADH:ubiquinone oxidoreductase subunit A8 (ndufa8) |
| Dissostichus_mawsoni_GLEAN_10012358 | 0.402703 | 0.004352 | M_034134830.1 | X-ray repair complementing defective repair in Chinese hamster cells 6 (xrcc6) |
| Dissostichus_mawsoni_GLEAN_10019941 | 0.297599 | 0.004708 | XM_034124221.1 | glutamate receptor, ionotropic, N-methyl D-aspartate-associated protein 1a (glutamate binding) (grinaa) |
| Dissostichus_mawsoni_GLEAN_10015214 | 0.436853 | 0.004929 | XM_034208559.1 | proline dehydrogenase (oxidase) 1a (prodha) |
| Dissostichus_mawsoni_GLEAN_10018966 | 0.218639 | 0.004929 | XM_034121395.1 | PRELI domain containing 1a (prelid1a) |
| Dissostichus_mawsoni_GLEAN_10009203 | 0.286744 | 0.004929 | XM_034128012.1 | MET transcriptional regulator MACC1 (macc1) |
| Dissostichus_mawsoni_GLEAN_10002236 | 0.441756 | 0.005078 | XM_051399048.1 | adaptor related protein complex 2 subunit mu 1b |
| Dissostichus_mawsoni_GLEAN_10017323 | 0.367278 | 0.006279 | XM_034151467.1 | centromere protein L (cenpl) |
| Dissostichus_mawsoni_GLEAN_10007477 | 0.354615 | 0.006574 | XM_034117264.1 | monoacylglycerol O-acyltransferase 2 (mogat2) |
| Dissostichus_mawsoni_GLEAN_10008119 | 0.161386 | 0.006875 | XM_034113197.1 | spartin b (spartb) |
| Dissostichus_mawsoni_GLEAN_10018127 | 0.474885 | 0.006875 | XM_034107938.1 | 60S ribosomal protein L35-like |
| Dissostichus_mawsoni_GLEAN_10022510 | 0.009356 | 0.006875 | XM_034217397.1 | sorbitol dehydrogenase (sord) |
| Dissostichus_mawsoni_GLEAN_10007278 | 0.581342 | 0.007667 | XM_034152940.1 | kinesin-1 heavy chain-like |
| Dissostichus_mawsoni_GLEAN_10008004 | 0.418844 | 0.007787 | XM_010768481.1 | cysteinyl-tRNA synthetase (cars) |
| Dissostichus_mawsoni_GLEAN_10022273 | 0.001679 | 0.007787 | XM_034105095.1 | DEAD (Asp-Glu-Ala-Asp) box polypeptide 28 (ddx28) |
| Dissostichus_mawsoni_GLEAN_10007020 | 0.135192 | 0.009051 | XM_034201489.1 | phospholipase A1 member A (pla1a) |
| Dissostichus_mawsoni_GLEAN_10017445 | 0.490877 | 0.010291 | XM_034147133.1 | mucolipin 3b (mcoln3b) |
| Dissostichus_mawsoni_GLEAN_10004969 | 0.295101 | 0.010921 | XM_034141492.1 | nedd4 binding protein 1 (n4bp1) |
| Dissostichus_mawsoni_GLEAN_10009620 | 0.037996 | 0.011044 | XM_034129071.1 | cyclin-dependent kinase inhibitor 3 (cdkn3) |
| Dissostichus_mawsoni_GLEAN_10006201 | 0.448427 | 0.011537 | XM_034212440.1 | cytosolic thiouridylase subunit 1 homolog (S. pombe) (ctu1) |
| Dissostichus_mawsoni_GLEAN_10013665 | 0.406777 | 0.011671 | XM_010784107.1 | zinc finger protein 362 (znf362) |
| Dissostichus_mawsoni_GLEAN_10001281 | 0.0103 | 0.013479 | XM_034150949.1 | F-box and leucine-rich repeat protein 5 (fbxl5) |
| Dissostichus_mawsoni_GLEAN_10007807 | 0.018312 | 0.014085 | XM_010775273.1 | RNA pseudouridylate synthase domain containing 4 (rpusd4) |
| Dissostichus_mawsoni_GLEAN_10012514 | 0.001804 | 0.014085 | XM_034138277.1 | zinc finger and BTB domain containing 33 (zbtb33) |
| Dissostichus_mawsoni_GLEAN_10001397 | 0.195695 | 0.014085 | XM_034141708.1 | CD82 antigen-like |
| Dissostichus_mawsoni_GLEAN_10001938 | 0.235951 | 0.01678 | XM_034208392.1 | mitochondrial ribosomal protein L41 (mrpl41) |
| Dissostichus_mawsoni_GLEAN_10013783 | 0.159571 | 0.018191 | XM_034153230.1 | methyltransferase like 17 (mettl17) |
| Dissostichus_mawsoni_GLEAN_10007593 | 0.439896 | 0.019517 | XM_034232058.1 | kidney mitochondrial carrier protein 1-like |
| Dissostichus_mawsoni_GLEAN_10007742 | 0.073486 | 0.019517 | XM_034223807.1 | poly (ADP-ribose) polymerase family, member 3 (parp3) |
| Dissostichus_mawsoni_GLEAN_10017340 | 0.605152 | 0.019517 | XM_034151404.1 | male-specific lethal 3 homolog |
| Dissostichus_mawsoni_GLEAN_10015497 | 0.002142 | 0.020751 | XM_034213415.1 | DnaJ (Hsp40) homolog, subfamily C, member 3a (dnajc3a) |
| Dissostichus_mawsoni_GLEAN_10015612 | 0.29857 | 0.020963 | XM_034219453.1 | cytochrome b-c1 complex subunit 2, mitochondrial |
| Dissostichus_mawsoni_GLEAN_10018020 | 0.455451 | 0.022022 | XM_034122783.1 | DnaJ heat shock protein family (Hsp40) member C21 (dnajc21) |
| Dissostichus_mawsoni_GLEAN_10012161 | 0.337432 | 0.022022 | XM_034126434.1 | PRA1 family protein 3-like |
| Dissostichus_mawsoni_GLEAN_10012140 | 0.340456 | 0.022387 | XM_034113619.1 | methyltransferase like 22 (mettl22) |
| Dissostichus_mawsoni_GLEAN_10015333 | 0.548783 | 0.023321 | XM_034076685.1 | family with sequence similarity 214 member A (fam214a) |
| Dissostichus_mawsoni_GLEAN_10015895 | 0.14549 | 0.024782 | XM_034141281.1 | ubiquitin-conjugating enzyme E2 Q2-like |
| Dissostichus_mawsoni_GLEAN_10021009 | 0.333887 | 0.024782 | XM_033637367.1 | SS18 subunit of BAF chromatin remodeling complex (ss18) |
| Dissostichus_mawsoni_GLEAN_10022018 | 0.391056 | 0.024782 | XM_034120836.1 | ubiquitin like modifier activating enzyme 6 (uba6) |
| Dissostichus_mawsoni_GLEAN_10021078 | 0.50295 | 0.024782 | XM_034226153.1 | presenilin associated, rhomboid-like a (parla) |
| Dissostichus_mawsoni_GLEAN_10016163 | 0.000175 | 0.024782 | XM_034133641.1 | malonyl CoA:ACP acyltransferase (mitochondrial) (mcat) |
| Dissostichus_mawsoni_GLEAN_10009230 | 0 | 0.025033 | XM_034127701.1 | gasdermin Eb (gsdmeb) |
| Dissostichus_mawsoni_GLEAN_10009161 | 0.512351 | 0.025183 | XM_034197091.1 | mitochondrial ribosomal protein S22 (mrps22) |
| Dissostichus_mawsoni_GLEAN_10017725 | 0.398202 | 0.026367 | XM_034110341.1 | mitochondrial pyruvate carrier 2b (mpc2b) |
| Dissostichus_mawsoni_GLEAN_10019090 | 0.114807 | 0.027083 | XM_034121001.1 | platelet-derived growth factor receptor-like (pdgfrl) |
| Dissostichus_mawsoni_GLEAN_10008811 | 0.299355 | 0.02784 | XM_034127477.1 | succinate dehydrogenase complex, subunit A, flavoprotein (Fp) (sdha) |
| Dissostichus_mawsoni_GLEAN_10016552 | 0 | 0.028486 | XM_010771134.1 | calcium binding and coiled-coil domain 2 (calcoco2) |
| Dissostichus_mawsoni_GLEAN_10017939 | 0.880011 | 0.030222 | XM_034123559.1 | nipped-B-like protein B |
| Dissostichus_mawsoni_GLEAN_10014373 | 0.00599 | 0.030222 | XM_034140151.1 | mitochondrial ribosomal protein S17 (mrps17) |
| Dissostichus_mawsoni_GLEAN_10017139 | 0.293739 | 0.03131 | XM_034221048.1 | RNA-binding protein with multiple splicing 2-like |
| Dissostichus_mawsoni_GLEAN_10000191 | 0.436765 | 0.03131 | XM_034209493.1 | caspase-9-like |
| Dissostichus_mawsoni_GLEAN_10016699 | 0.208664 | 0.03131 | XM_034238338.1 | arrestin domain containing 3a (arrdc3a) |
| Dissostichus_mawsoni_GLEAN_10018629 | 0.360521 | 0.031565 | XM_034218663.1 | parvin, beta (parvb) |
| Dissostichus_mawsoni_GLEAN_10011111 | 0.145154 | 0.031565 | XM_034112734.1 | colony stimulating factor 3 receptor (granulocyte) (csf3r) |
| Dissostichus_mawsoni_GLEAN_10013546 | 0.332866 | 0.031639 | XM_039782507.1 | breast cancer metastasis-suppressor 1-like protein-A |
| Dissostichus_mawsoni_GLEAN_10012381 | 0.224485 | 0.032574 | XM_034119240.1 | translocase of outer mitochondrial membrane 22 homolog (yeast) (tomm22) |
| Dissostichus_mawsoni_GLEAN_10009498 | 0.252862 | 0.033851 | XM_034127907.1 | aryl hydrocarbon receptor nuclear translocator-like |
| Dissostichus_mawsoni_GLEAN_10015887 | 0.293022 | 0.035715 | XM_034126361.1 | SAM domain and HD domain 1 (samhd1) |
| Dissostichus_mawsoni_GLEAN_10015568 | 0.002501 | 0.036024 | XM_034220016.1 | SRY-box transcription factor 9a (sox9a) |
| Dissostichus_mawsoni_GLEAN_10012043 | 0.217597 | 0.037505 | XM_034119704.1 | uroporphyrinogen III synthase (uros) |
| Dissostichus_mawsoni_GLEAN_10012816 | 0.231211 | 0.037607 | XM_034096140.1 | proteinase-activated receptor 1-like |
| Dissostichus_mawsoni_GLEAN_10019381 | 0.447788 | 0.03789 | XM_034139281.1 | protein phosphatase 4, regulatory subunit 1-like (ppp4r1l) |
| Dissostichus_mawsoni_GLEAN_10021854 | 0.012664 | 0.038304 | XM_034203511.1 | microsomal triglyceride transfer protein (mttp) |
| Dissostichus_mawsoni_GLEAN_10008560 | 0.692586 | 0.038352 | XM_034121758.1 | ribophorin II (rpn2) |
| Dissostichus_mawsoni_GLEAN_10011243 | 0.291512 | 0.038434 | XM_034216408.1 | retinol dehydrogenase 10a (rdh10a) |
| Dissostichus_mawsoni_GLEAN_10019911 | 0.012724 | 0.038751 | XM_034094007.1 | mitochondrial ribosomal protein L53 (mrpl53) |
| Dissostichus_mawsoni_GLEAN_10020163 | 0.385932 | 0.03954 | XM_010779990.1 | serpin A3-2-like |
| Dissostichus_mawsoni_GLEAN_10005343 | 0.501919 | 0.039728 | XM_034134139.1 | ankyrin repeat and SOCS box containing 1 (asb1) |
| Dissostichus_mawsoni_GLEAN_10003727 | 0.314678 | 0.040651 | XM_034225239.1 | ribosomal protein S30 (mrps30) |
| Dissostichus_mawsoni_GLEAN_10003624 | 0.504531 | 0.04213 | XM_034125605.1 | TMCO1/EMC3 family protein (zgc:86609) |
| Dissostichus_mawsoni_GLEAN_10007688 | 0.474402 | 0.04213 | XM_034092981.1 | early growth response 1 (egr1) |
| Dissostichus_mawsoni_GLEAN_10010739 | 0.000985 | 0.042738 | XM_034237286.1 | synaptosome associated protein 29 (snap29) |
| Dissostichus_mawsoni_GLEAN_10016153 | 0.088273 | 0.042905 | XM_034133532.1 | t-complex 11, testis-specific-like 2 (tcp11l2) |
| Dissostichus_mawsoni_GLEAN_10019142 | 0.523564 | 0.044371 | XM_034092394.1 | fam199x |
| Dissostichus_mawsoni_GLEAN_10020936 | 0.457201 | 0.044446 | XM_034137855.1 | synaptosome associated protein 47 (snap47) |
| Dissostichus_mawsoni_GLEAN_10022033 | 0.78905 | 0.044531 | XM_034120313.1 | ubiquitin-conjugating enzyme E2Kb (UBC1 homolog, yeast) (ube2kb) |
| Dissostichus_mawsoni_GLEAN_10007454 | 0.000542 | 0.045904 | XM_034126253.1 | SAC1 like phosphatidylinositide phosphatase a (sacm1la) |
| Dissostichus_mawsoni_GLEAN_10016071 | 0.589591 | 0.046819 | XM_034141925.1 | von Willebrand factor (vwf), mRNA |
| Dissostichus_mawsoni_GLEAN_10021312 | 0 | 0.046819 | XM_034231772.1 | platelet-activating factor receptor (ptafr) |
| Dissostichus_mawsoni_GLEAN_10010888 | 0.573864 | 0.048835 | XM_034120692.1 | N-acetylneuraminic acid synthase a (nansa) |
| Dissostichus_mawsoni_GLEAN_10016978 | 0.749255 | 0.048999 | XM_034227409.1 | G protein-coupled receptor 137Ba |
| Dissostichus_mawsoni_GLEAN_10009324 | 0.622627 | 0.049584 | XM_034152948.1 | GTP binding protein 4 (gtpbp4) |
| Dissostichus_mawsoni_GLEAN_10018990 | 0.543561 | 0.049661 | XM_034232554.1 | TNFAIP3 interacting protein 1 (tnip1) |

The *D. mawsoni* contig ID column shows the contig ID for the *D. mawsoni* member of each significant orthogroup based on the predicted peptide naming used in assembly from Chen et al. 2019

The K column shows the relative relaxation of selective pressure as determined by RELAX

The FDR column shows the multiple hypothesis corrected p-values that resulted for RELAX

The Accession column shows the accession number of the best blast hit from searching NCBI’s nt database for that gene

The gene description column shows the descriptive name for the orthogroup based on the BLAST search against NCBI’s nt database

**S Table 12:**

**Genes associated with GO terms in the Gene Expression Cluster**

| **Contig ID in *D. mawsoni*** | **Gene Name** | **GO Term(s)** |
| --- | --- | --- |
|  |  |  |
| Dissostichus_mawsoni_GLEAN_10001174 | signal transducer and activator of transcription 1a (stat1a) | GO:0044271, GO:0006355, GO:1903506, GO:2001141, GO:0009889, GO:0010556, GO:0019219, GO:0031326, GO:0051252, GO:2000112, GO:0031323, GO:0051171, GO:0080090, GO:0050794, GO:0009058, GO:0009059, GO:0034645, GO:0044249, GO:1901576, GO:0010468, GO:0060255, GO:0019222, GO:0006351, GO:0018130, GO:0019438, GO:0032774, GO:0034654, GO:0097659, GO:1901362, GO:0050789, GO:0044260, GO:0010467, GO:0065007 |
| Dissostichus_mawsoni_GLEAN_10003175 | cyclin T2 (ccnt2) | GO:0044271, GO:0006355, GO:1903506, GO:2001141, GO:0009889, GO:0010556, GO:0019219, GO:0031326, GO:0051252, GO:2000112, GO:0031323, GO:0051171, GO:0080090, GO:0050794, GO:0009058, GO:0009059, GO:0034645, GO:0044249, GO:1901576, GO:0010468, GO:0060255, GO:0019222, GO:0006351, GO:0018130, GO:0019438, GO:0032774, GO:0034654, GO:0097659, GO:1901362, GO:0050789, GO:0044260, GO:0010467, GO:0065007 |
| Dissostichus_mawsoni_GLEAN_10005600 | zinc finger, CCCH-type with G patch domain (zgpat) | GO:0044271, GO:0006355, GO:1903506, GO:2001141, GO:0009889, GO:0010556, GO:0019219, GO:0031326, GO:0051252, GO:2000112, GO:0031323, GO:0051171, GO:0080090, GO:0050794, GO:0009058, GO:0009059, GO:0034645, GO:0044249, GO:1901576, GO:0010468, GO:0060255, GO:0019222, GO:0006351, GO:0018130, GO:0019438, GO:0032774, GO:0034654, GO:0097659, GO:1901362, GO:0050789, GO:0044260, GO:0010467, GO:0065007 |
| Dissostichus_mawsoni_GLEAN_10007894 | E74-like ETS transcription factor 1 (elf1) | GO:0044271, GO:0006355, GO:1903506, GO:2001141, GO:0009889, GO:0010556, GO:0019219, GO:0031326, GO:0051252, GO:2000112, GO:0031323, GO:0051171, GO:0080090, GO:0050794, GO:0009058, GO:0009059, GO:0034645, GO:0044249, GO:1901576, GO:0010468, GO:0060255, GO:0019222, GO:0006351, GO:0018130, GO:0019438, GO:0032774, GO:0034654, GO:0097659, GO:1901362, GO:0050789, GO:0044260, GO:0010467, GO:0065007 |
| Dissostichus_mawsoni_GLEAN_10008024 | BRCA1 associated protein-1 (ubiquitin carboxy-terminal hydrolase) (bap1) | GO:0044271, GO:0006355, GO:1903506, GO:2001141, GO:0009889, GO:0010556, GO:0019219, GO:0031326, GO:0051252, GO:2000112, GO:0031323, GO:0051171, GO:0080090, GO:0050794, GO:0009058, GO:0009059, GO:0034645, GO:0044249, GO:1901576, GO:0010468, GO:0060255, GO:0019222, GO:0006351, GO:0018130, GO:0019438, GO:0032774, GO:0034654, GO:0097659, GO:1901362, GO:0050789, GO:0044260, GO:0010467, GO:0065007 |
| Dissostichus_mawsoni_GLEAN_10010316 | forkhead box A2 (foxa2) | GO:0044271, GO:0006355, GO:1903506, GO:2001141, GO:0009889, GO:0010556, GO:0019219, GO:0031326, GO:0051252, GO:2000112, GO:0031323, GO:0051171, GO:0080090, GO:0050794, GO:0009058, GO:0009059, GO:0034645, GO:0044249, GO:1901576, GO:0010468, GO:0060255, GO:0019222, GO:0006351, GO:0018130, GO:0019438, GO:0032774, GO:0034654, GO:0097659, GO:1901362, GO:0050789, GO:0044260, GO:0010467, GO:0065007 |
| Dissostichus_mawsoni_GLEAN_10010728 | UTP15 small subunit processome component (utp15) | GO:0044271, GO:0006355, GO:1903506, GO:2001141, GO:0009889, GO:0010556, GO:0019219, GO:0031326, GO:0051252, GO:2000112, GO:0031323, GO:0051171, GO:0080090, GO:0050794, GO:0009058, GO:0009059, GO:0034645, GO:0044249, GO:1901576, GO:0010468, GO:0060255, GO:0019222, GO:0006351, GO:0018130, GO:0019438, GO:0032774, GO:0034654, GO:0097659, GO:1901362, GO:0050789, GO:0044260, GO:0010467, GO:0065007 |
| Dissostichus_mawsoni_GLEAN_10009914 | mediator complex subunit 23 (med23) | GO:0044271, GO:0006355, GO:1903506, GO:2001141, GO:0009889, GO:0010556, GO:0019219, GO:0031326, GO:0051252, GO:2000112, GO:0031323, GO:0051171, GO:0080090, GO:0050794, GO:0009058, GO:0009059, GO:0034645, GO:0044249, GO:1901576, GO:0010468, GO:0060255, GO:0019222, GO:0006351, GO:0018130, GO:0019438, GO:0032774, GO:0034654, GO:0097659, GO:1901362, GO:0050789, GO:0044260, GO:0010467, GO:0065007 |
| Dissostichus_mawsoni_GLEAN_10010870 | programmed cell death 4b (pdcd4b) | GO:0044271, GO:0006355, GO:1903506, GO:2001141, GO:0009889, GO:0010556, GO:0019219, GO:0031326, GO:0051252, GO:2000112, GO:0031323, GO:0051171, GO:0080090, GO:0050794, GO:0009058, GO:0009059, GO:0034645, GO:0044249, GO:1901576, GO:0010468, GO:0060255, GO:0019222, GO:0006351, GO:0018130, GO:0019438, GO:0032774, GO:0034654, GO:0097659, GO:1901362, GO:0050789, GO:0044260, GO:0010467, GO:0065007 |
| Dissostichus_mawsoni_GLEAN_10012794 | strawberry notch homolog 1 (sbno1) | GO:0044271, GO:0006355, GO:1903506, GO:2001141, GO:0009889, GO:0010556, GO:0019219, GO:0031326, GO:0051252, GO:2000112, GO:0031323, GO:0051171, GO:0080090, GO:0050794, GO:0009058, GO:0009059, GO:0034645, GO:0044249, GO:1901576, GO:0010468, GO:0060255, GO:0019222, GO:0006351, GO:0018130, GO:0019438, GO:0032774, GO:0034654, GO:0097659, GO:1901362, GO:0050789, GO:0044260, GO:0010467, GO:0065007 |
| Dissostichus_mawsoni_GLEAN_10010743 | inhibitor of nuclear factor kappa B kinase subunit beta (ikbkb) | GO:0044271, GO:0006355, GO:1903506, GO:2001141, GO:0009889, GO:0010556, GO:0019219, GO:0031326, GO:0051252, GO:2000112, GO:0031323, GO:0051171, GO:0080090, GO:0050794, GO:0009058, GO:0009059, GO:0034645, GO:0044249, GO:1901576, GO:0010468, GO:0060255, GO:0019222, GO:0006351, GO:0018130, GO:0019438, GO:0032774, GO:0034654, GO:0097659, GO:1901362, GO:0050789, GO:0044260, GO:0010467, GO:0065007 |
| Dissostichus_mawsoni_GLEAN_10013547 | NF-kappa-B inhibitor alpha-like | GO:0044271, GO:0006355, GO:1903506, GO:2001141, GO:0009889, GO:0010556, GO:0019219, GO:0031326, GO:0051252, GO:2000112, GO:0031323, GO:0051171, GO:0080090, GO:0050794, GO:0009058, GO:0009059, GO:0034645, GO:0044249, GO:1901576, GO:0010468, GO:0060255, GO:0019222, GO:0006351, GO:0018130, GO:0019438, GO:0032774, GO:0034654, GO:0097659, GO:1901362, GO:0050789, GO:0044260, GO:0010467, GO:0065007 |
| Dissostichus_mawsoni_GLEAN_10015766 | calcium-responsive transactivator-like | GO:0044271, GO:0006355, GO:1903506, GO:2001141, GO:0009889, GO:0010556, GO:0019219, GO:0031326, GO:0051252, GO:2000112, GO:0031323, GO:0051171, GO:0080090, GO:0050794, GO:0009058, GO:0009059, GO:0034645, GO:0044249, GO:1901576, GO:0010468, GO:0060255, GO:0019222, GO:0006351, GO:0018130, GO:0019438, GO:0032774, GO:0034654, GO:0097659, GO:1901362, GO:0050789, GO:0044260, GO:0010467, GO:0065007 |
| Dissostichus_mawsoni_GLEAN_10012257 | N-acetyllactosaminide beta-1,3-N-acetylglucosaminyltransferase 3-like | GO:0009058, GO:0009059, GO:0034645, GO:0044249, GO:1901576, GO:0044260 |
| Dissostichus_mawsoni_GLEAN_10016863 | THO complex 5 (thoc5) | GO:0044271, GO:0006355, GO:1903506, GO:2001141, GO:0009889, GO:0010556, GO:0019219, GO:0031326, GO:0051252, GO:2000112, GO:0031323, GO:0051171, GO:0080090, GO:0050794, GO:0009058, GO:0009059, GO:0034645, GO:0044249, GO:1901576, GO:0010468, GO:0060255, GO:0019222, GO:0006351, GO:0018130, GO:0019438, GO:0032774, GO:0034654, GO:0097659, GO:1901362, GO:0050789, GO:0044260, GO:0010467, GO:0065007 |
| Dissostichus_mawsoni_GLEAN_10018459 | CASP8 and FADD-like apoptosis regulator | GO:0044271, GO:0006355, GO:1903506, GO:2001141, GO:0009889, GO:0010556, GO:0019219, GO:0031326, GO:0051252, GO:2000112, GO:0031323, GO:0051171, GO:0080090, GO:0050794, GO:0009058, GO:0009059, GO:0034645, GO:0044249, GO:1901576, GO:0010468, GO:0060255, GO:0019222, GO:0006351, GO:0018130, GO:0019438, GO:0032774, GO:0034654, GO:0097659, GO:1901362, GO:0050789, GO:0044260, GO:0010467, GO:0065007 |
| Dissostichus_mawsoni_GLEAN_10015284 | iroquois homeobox 3a (irx3a) | GO:0044271, GO:0006355, GO:1903506, GO:2001141, GO:0009889, GO:0010556, GO:0019219, GO:0031326, GO:0051252, GO:2000112, GO:0031323, GO:0051171, GO:0080090, GO:0050794, GO:0009058, GO:0009059, GO:0034645, GO:0044249, GO:1901576, GO:0010468, GO:0060255, GO:0019222, GO:0006351, GO:0018130, GO:0019438, GO:0032774, GO:0034654, GO:0097659, GO:1901362, GO:0050789, GO:0044260, GO:0010467, GO:0065007 |
| Dissostichus_mawsoni_GLEAN_10018841 | MAF1 homolog (S. cerevisiae) (maf1) | GO:0044271, GO:0006355, GO:1903506, GO:2001141, GO:0009889, GO:0010556, GO:0019219, GO:0031326, GO:0051252, GO:2000112, GO:0031323, GO:0051171, GO:0080090, GO:0050794, GO:0009058, GO:0009059, GO:0034645, GO:0044249, GO:1901576, GO:0010468, GO:0060255, GO:0019222, GO:0006351, GO:0018130, GO:0019438, GO:0032774, GO:0034654, GO:0097659, GO:1901362, GO:0050789, GO:0044260, GO:0010467, GO:0065007 |
| Dissostichus_mawsoni_GLEAN_10019524 | activating transcription factor 1 (atf1) | GO:0044271, GO:0006355, GO:1903506, GO:2001141, GO:0009889, GO:0010556, GO:0019219, GO:0031326, GO:0051252, GO:2000112, GO:0031323, GO:0051171, GO:0080090, GO:0050794, GO:0009058, GO:0009059, GO:0034645, GO:0044249, GO:1901576, GO:0010468, GO:0060255, GO:0019222, GO:0006351, GO:0018130, GO:0019438, GO:0032774, GO:0034654, GO:0097659, GO:1901362, GO:0050789, GO:0044260, GO:0010467, GO:0065007 |
| Dissostichus_mawsoni_GLEAN_10018274 | interferon regulatory factor 3 (irf3) | GO:0044271, GO:0006355, GO:1903506, GO:2001141, GO:0009889, GO:0010556, GO:0019219, GO:0031326, GO:0051252, GO:2000112, GO:0031323, GO:0051171, GO:0080090, GO:0050794, GO:0009058, GO:0009059, GO:0034645, GO:0044249, GO:1901576, GO:0010468, GO:0060255, GO:0019222, GO:0006351, GO:0018130, GO:0019438, GO:0032774, GO:0034654, GO:0097659, GO:1901362, GO:0050789, GO:0044260, GO:0010467, GO:0065007 |
| Dissostichus_mawsoni_GLEAN_10018954 | ectodysplasin A (eda) | GO:0044271, GO:0006355, GO:1903506, GO:2001141, GO:0009889, GO:0010556, GO:0019219, GO:0031326, GO:0051252, GO:2000112, GO:0031323, GO:0051171, GO:0080090, GO:0050794, GO:0009058, GO:0009059, GO:0034645, GO:0044249, GO:1901576, GO:0010468, GO:0060255, GO:0019222, GO:0006351, GO:0018130, GO:0019438, GO:0032774, GO:0034654, GO:0097659, GO:1901362, GO:0050789, GO:0044260, GO:0010467, GO:0065007 |
| Dissostichus_mawsoni_GLEAN_10020323 | histidine triad nucleotide binding protein 1 (hint1) | GO:0044271, GO:0006355, GO:1903506, GO:2001141, GO:0009889, GO:0010556, GO:0019219, GO:0031326, GO:0051252, GO:2000112, GO:0031323, GO:0051171, GO:0080090, GO:0050794, GO:0009058, GO:0009059, GO:0034645, GO:0044249, GO:1901576, GO:0010468, GO:0060255, GO:0019222, GO:0006351, GO:0018130, GO:0019438, GO:0032774, GO:0034654, GO:0097659, GO:1901362, GO:0050789, GO:0044260, GO:0010467, GO:0065007 |
| Dissostichus_mawsoni_GLEAN_10020593 | glutamate-cysteine ligase, catalytic subunit (gclc) | GO:0044271, GO:0006355, GO:1903506, GO:2001141, GO:0009889, GO:0010556, GO:0019219, GO:0031326, GO:0051252, GO:2000112, GO:0031323, GO:0051171, GO:0080090, GO:0050794, GO:0009058, GO:0009059, GO:0034645, GO:0044249, GO:1901576, GO:0010468, GO:0060255, GO:0019222, GO:0006351, GO:0018130, GO:0019438, GO:0032774, GO:0034654, GO:0097659, GO:1901362, GO:0050789, GO:0044260, GO:0010467, GO:0065007 |
| Dissostichus_mawsoni_GLEAN_10020585 | microcephalin 1 (mcph1) | GO:0044271, GO:0006355, GO:1903506, GO:2001141, GO:0009889, GO:0010556, GO:0019219, GO:0031326, GO:0051252, GO:2000112, GO:0031323, GO:0051171, GO:0080090, GO:0050794, GO:0009058, GO:0009059, GO:0034645, GO:0044249, GO:1901576, GO:0010468, GO:0060255, GO:0019222, GO:0006351, GO:0018130, GO:0019438, GO:0032774, GO:0034654, GO:0097659, GO:1901362, GO:0050789, GO:0044260, GO:0010467, GO:0065007 |
| Dissostichus_mawsoni_GLEAN_10020700 | PR domain containing 4 (prdm4) | GO:0044271, GO:0009058, GO:0009059, GO:0034645, GO:0044249, GO:1901576, GO:0006351, GO:0018130, GO:0019438, GO:0032774, GO:0034654, GO:0097659, GO:1901362, GO:0044260, GO:0010467 |
| Dissostichus_mawsoni_GLEAN_10001714 | K(lysine) acetyltransferase 8 (kat8) | GO:0044271, GO:0006355, GO:1903506, GO:2001141, GO:0009889, GO:0010556, GO:0019219, GO:0031326, GO:0051252, GO:2000112, GO:0031323, GO:0051171, GO:0080090, GO:0050794, GO:0009058, GO:0009059, GO:0034645, GO:0044249, GO:1901576, GO:0010468, GO:0060255, GO:0019222, GO:0006351, GO:0018130, GO:0019438, GO:0032774, GO:0034654, GO:0097659, GO:1901362, GO:0050789, GO:0044260, GO:0010467, GO:0065007 |
| Dissostichus_mawsoni_GLEAN_10005573 | upstream stimulatory factor 1-like | GO:0044271, GO:0006355, GO:1903506, GO:2001141, GO:0009889, GO:0010556, GO:0019219, GO:0031326, GO:0051252, GO:2000112, GO:0031323, GO:0051171, GO:0080090, GO:0050794, GO:0009058, GO:0009059, GO:0034645, GO:0044249, GO:1901576, GO:0010468, GO:0060255, GO:0019222, GO:0006351, GO:0018130, GO:0019438, GO:0032774, GO:0034654, GO:0097659, GO:1901362, GO:0050789, GO:0044260, GO:0010467, GO:0065007 |
| Dissostichus_mawsoni_GLEAN_10007266 | serine/threonine-protein kinase pim-3-like | GO:0044271, GO:0009058, GO:0009059, GO:0034645, GO:0044249, GO:1901576, GO:0044260, GO:0010467 |
| Dissostichus_mawsoni_GLEAN_10008599 | alpha-1,3-mannosyl-glycoprotein 4-beta-N-acetylglucosaminyltransferase C-like | GO:0009058, GO:0009059, GO:0034645, GO:0044249, GO:1901576, GO:0044260 |
| Dissostichus_mawsoni_GLEAN_10011174 | translation initiation factor 3 subunit H | GO:0044271, GO:0009058, GO:0009059, GO:0034645, GO:0044249, GO:1901576, GO:0044260, GO:0010467 |
| Dissostichus_mawsoni_GLEAN_10001377 | nuclear factor of activated T-cells, cytoplasmic 3-like | GO:0044271, GO:0006355, GO:1903506, GO:2001141, GO:0009889, GO:0010556, GO:0019219, GO:0031326, GO:0051252, GO:2000112, GO:0031323, GO:0051171, GO:0080090, GO:0050794, GO:0009058, GO:0009059, GO:0034645, GO:0044249, GO:1901576, GO:0010468, GO:0060255, GO:0019222, GO:0006351, GO:0018130, GO:0019438, GO:0032774, GO:0034654, GO:0097659, GO:1901362, GO:0050789, GO:0044260, GO:0010467, GO:0065007 |
| Dissostichus_mawsoni_GLEAN_10004964 | alanyl-tRNA synthetase 1 (aars1) | GO:0044271, GO:0006355, GO:1903506, GO:2001141, GO:0009889, GO:0010556, GO:0019219, GO:0031326, GO:0051252, GO:2000112, GO:0031323, GO:0051171, GO:0080090, GO:0050794, GO:0009058, GO:0009059, GO:0034645, GO:0044249, GO:1901576, GO:0010468, GO:0060255, GO:0019222, GO:0006351, GO:0018130, GO:0019438, GO:0032774, GO:0034654, GO:0097659, GO:1901362, GO:0050789, GO:0044260, GO:0010467, GO:0065007 |
| Dissostichus_mawsoni_GLEAN_10005625 | regulatory factor X, 1b (influences HLA class II expression) (rfx1b) | GO:0044271, GO:0006355, GO:1903506, GO:2001141, GO:0009889, GO:0010556, GO:0019219, GO:0031326, GO:0051252, GO:2000112, GO:0031323, GO:0051171, GO:0080090, GO:0050794, GO:0009058, GO:0009059, GO:0034645, GO:0044249, GO:1901576, GO:0010468, GO:0060255, GO:0019222, GO:0006351, GO:0018130, GO:0019438, GO:0032774, GO:0034654, GO:0097659, GO:1901362, GO:0050789, GO:0044260, GO:0010467, GO:0065007 |
| Dissostichus_mawsoni_GLEAN_10018578 | iron-responsive element binding protein 2 (ireb2) | GO:0065007 |
| Dissostichus_mawsoni_GLEAN_10018296 | kinesin family member 22 (kif22) | GO:0044260 |
| Dissostichus_mawsoni_GLEAN_10020877 | transcription elongation factor A (SII), 1 (tcea1) | GO:0044271, GO:0006355, GO:1903506, GO:2001141, GO:0009889, GO:0010556, GO:0019219, GO:0031326, GO:0051252, GO:2000112, GO:0031323, GO:0051171, GO:0080090, GO:0050794, GO:0009058, GO:0009059, GO:0034645, GO:0044249, GO:1901576, GO:0010468, GO:0060255, GO:0019222, GO:0006351, GO:0018130, GO:0019438, GO:0032774, GO:0034654, GO:0097659, GO:1901362, GO:0050789, GO:0044260, GO:0010467, GO:0065007 |
| Dissostichus_mawsoni_GLEAN_10019398 | replication termination factor 2 (rtf2) | GO:0009058, GO:0009059, GO:0034645, GO:0044249, GO:1901576, GO:0044260 |

The *D. mawsoni* contig ID column shows the contig ID for the *D. mawsoni* member of each significant orthogroup based on the predicted peptide naming used in assembly from Chen et al. 2019

The gene description column shows the descriptive name for the orthogroup based on the BLAST search against NCBI’s nt database.

The GOTerm(s) column shows the enriched Gene Ontology Terms that each contig is associated with.

**S Table 13:**

**Genes associated with GO term in the Cell Cycle Cluster**

| **Contig ID in *D. mawsoni*** | **Gene Name** | **GO Term(s)** |
| --- | --- | --- |
|  |  |  |
| Dissostichus_mawsoni_GLEAN_10004584 | dual specificity protein phosphatase CDC14AB-like | GO:0022402, GO:0007049, GO:0000278, GO:0140014, GO:1903047 |
| Dissostichus_mawsoni_GLEAN_10004700 | UF2 component of NDC80 kinetochore complex (nuf2) | GO:0022402, GO:0007049, GO:0000278, GO:0140014, GO:1903047 |
| Dissostichus_mawsoni_GLEAN_10010743 | inhibitor of nuclear factor kappa B kinase subunit beta (ikbkb) | GO:0022402, GO:0007049 |
| Dissostichus_mawsoni_GLEAN_10011101 | guanine nucleotide binding protein-like 2 (nucleolar) (gnl2) | GO:0022402, GO:0007049, GO:0000278, GO:0140014, GO:1903047 |
| Dissostichus_mawsoni_GLEAN_10018029 | developmentally regulated GTP binding protein 1 (drg1) | GO:0022402, GO:0007049, GO:0000278, GO:0140014, GO:1903047 |
| Dissostichus_mawsoni_GLEAN_10012734 | SDA1 domain containing 1 (sdad1) | GO:0007049, GO:0000278 |
| Dissostichus_mawsoni_GLEAN_10018296 | kinesin family member 22 (kif22) | GO:0022402, GO:0007049, GO:0000278, GO:0140014, GO:1903047 |
| Dissostichus_mawsoni_GLEAN_10016197 | guanine nucleotide binding protein (G protein), alpha inhibiting activity polypeptide 1 (gnai1) | GO:0007049, GO:0000278 |
| Dissostichus_mawsoni_GLEAN_10019981 | programmed cell death 6 interacting protein (pdcd6ip) | GO:0022402, GO:0007049, GO:0000278 |
| Dissostichus_mawsoni_GLEAN_10019398 | replication termination factor 2 (rtf2) | GO:0007049, GO:0000278 |
| Dissostichus_mawsoni_GLEAN_10019851 | proline rich mitotic checkpoint control factor (prcc) | GO:0007049, GO:0000278 |
| Dissostichus_mawsoni_GLEAN_10020585 | microcephalin 1 (mcph1) | GO:0007049, GO:0000278 |

The *D. mawsoni* contig ID column shows the contig ID for the *D. mawsoni* member of each significant orthogroup based on the predicted peptide naming used in assembly from Chen et al. 2019

The gene description column shows the descriptive name for the orthogroup based on the BLAST search against NCBI’s nt database.

The GOTerm(s) column shows the enriched Gene Ontology Terms that each contig is associated with.

**S Table 14:**

**Genes associated with GO term in the Cell Adhesion Cluster**

| **Contig ID in *D. mawsoni*** | **Gene Name** | **GO Term(s)** |
| --- | --- | --- |
|  |  |  |
| Dissostichus_mawsoni_GLEAN_10001356 | B-cadherin-like | GO:0007155, GO:0022610 |
| Dissostichus_mawsoni_GLEAN_10006966 | CD9 molecule a (cd9a) | GO:0007155, GO:0022610 |
| Dissostichus_mawsoni_GLEAN_10007976 | epithelial cell adhesion molecule (epcam) | GO:0007155, GO:0022610 |
| Dissostichus_mawsoni_GLEAN_10010332 | LCK proto-oncogene, Src family tyrosine kinase (lck) | GO:0007155, GO:0022610 |
| Dissostichus_mawsoni_GLEAN_10013325 | torsin family 1 (tor1) | GO:0007155, GO:0022610 |
| Dissostichus_mawsoni_GLEAN_10014488 | transmembrane protein 47-like | GO:0007155, GO:0022610 |
| Dissostichus_mawsoni_GLEAN_10018348 | attractin-like 1b (atrnl1b) | GO:0007155, GO:0022610 |
| Dissostichus_mawsoni_GLEAN_10018996 | catenin (cadherin-associated protein), alpha 1 (ctnna1) | GO:0007155, GO:0022610 |
| Dissostichus_mawsoni_GLEAN_10021003 | desmocollin 2 like (dsc2l) | GO:0007155, GO:0022610 |

The *D. mawsoni* contig ID column shows the contig ID for the *D. mawsoni* member of each significant orthogroup based on the predicted peptide naming used in assembly from Chen et al. 2019

The gene description column shows the descriptive name for the orthogroup based on the BLAST search against NCBI’s nt database.

The GOTerm(s) column shows the enriched Gene Ontology Terms that each contig is associated with.

**S Table 15:**

**Genes associated with GO term in the Mitochondrion Cluster**

| **Contig ID in *D. mawsoni*** | **Gene Name** | **GO Term(s)** |
| --- | --- | --- |
|  |  |  |
| Dissostichus_mawsoni_GLEAN_10001938 | mitochondrial ribosomal protein L41 (mrpl41) | GO:0005739 |
| Dissostichus_mawsoni_GLEAN_10003727 | ribosomal protein S30 (mrps30) | GO:0005739 |
| Dissostichus_mawsoni_GLEAN_10008119 | spartin b (spartb) | GO:0005739 |
| Dissostichus_mawsoni_GLEAN_10008811 | succinate dehydrogenase complex, subunit A, flavoprotein (Fp) (sdha) | GO:0005739 |
| Dissostichus_mawsoni_GLEAN_10009161 | mitochondrial ribosomal protein S22 (mrps22) | GO:0005739 |
| Dissostichus_mawsoni_GLEAN_10009203 | MET transcriptional regulator MACC1 (macc1) | GO:0005739 |
| Dissostichus_mawsoni_GLEAN_10012381 | translocase of outer mitochondrial membrane 22 homolog (yeast) (tomm22) | GO:0005739 |
| Dissostichus_mawsoni_GLEAN_10013783 | methyltransferase like 17 (mettl17) | GO:0005739 |
| Dissostichus_mawsoni_GLEAN_10014373 | mitochondrial ribosomal protein S17 (mrps17) | GO:0005739 |
| Dissostichus_mawsoni_GLEAN_10015214 | proline dehydrogenase (oxidase) 1a (prodha) | GO:0005739 |
| Dissostichus_mawsoni_GLEAN_10015612 | cytochrome b-c1 complex subunit 2, mitochondrial | GO:0005739 |
| Dissostichus_mawsoni_GLEAN_10016163 | malonyl CoA:ACP acyltransferase (mitochondrial) (mcat) | GO:0005739 |
| Dissostichus_mawsoni_GLEAN_10016268 | cytochrome c oxidase assembly factor 7 | GO:0005739 |
| Dissostichus_mawsoni_GLEAN_10017141 | solute carrier family 25 member 44-like | GO:0005739 |
| Dissostichus_mawsoni_GLEAN_10017600 | FAST kinase domains 1 (fastkd1) | GO:0005739 |
| Dissostichus_mawsoni_GLEAN_10017725 | mitochondrial pyruvate carrier 2b (mpc2b) | GO:0005739 |
| Dissostichus_mawsoni_GLEAN_10018042 | nicotinamide nucleotide transhydrogenase (nnt) | GO:0005739 |
| Dissostichus_mawsoni_GLEAN_10018048 | NADH:ubiquinone oxidoreductase subunit A8 (ndufa8) | GO:0005739 |
| Dissostichus_mawsoni_GLEAN_10018318 | translational activator of cytochrome c oxidase 1-like | GO:0005739 |
| Dissostichus_mawsoni_GLEAN_10018966 | PRELI domain containing 1a (prelid1a) | GO:0005739 |
| Dissostichus_mawsoni_GLEAN_10019911 | mitochondrial ribosomal protein L53 (mrpl53) | GO:0005739 |
| Dissostichus_mawsoni_GLEAN_10021925 | transcription elongation factor, mitochondrial (tefm) | GO:0005739 |
| Dissostichus_mawsoni_GLEAN_10022273 | DEAD (Asp-Glu-Ala-Asp) box polypeptide 28 (ddx28) | GO:0005739 |

The *D. mawsoni* contig ID column shows the contig ID for the *D. mawsoni* member of each significant orthogroup based on the predicted peptide naming used in assembly from Chen et al. 2019

The gene description column shows the descriptive name for the orthogroup based on the BLAST search against NCBI’s nt database.

The GOTerm(s) column shows the enriched Gene Ontology Terms that each contig is associated with.
