# Supplementary material for: Positive and Relaxed Selective Pressures Have Both Strongly Influenced the Evolution of Cryonotothenioid Fishes during Their Radiation in the Freezing Southern Ocean
Source: Genome Biol Evol. 2023 Mar 23;15(4):evad049. doi: 10.1093/gbe/evad049 (PMC10078794; doi:10.1093/gbe/evad049)

**Positive and relaxed selective pressures have both strongly influenced the evolution of cryonotothenioid fishes during their radiation in the freezing Southern Ocean**

**Supplementary Figures**

**SECTION** **PAGE**

S Figure 1 ………………………………………………………………………………………… 2

Phylogenetic tree used in selective pressure analyses

S Figure 2 ………………………………………………………………………………………… 3

Tissue completeness of each notothenioid species’ transcriptome

S Figure 3 ………………………………………………………………………………………… 4

Comparison of genomic and transcriptomic derived predicted peptides using BUSCO for three cryonotothenioid species

**S Figure 1:**

**Phylogenetic reconstruction of the species used in this study**


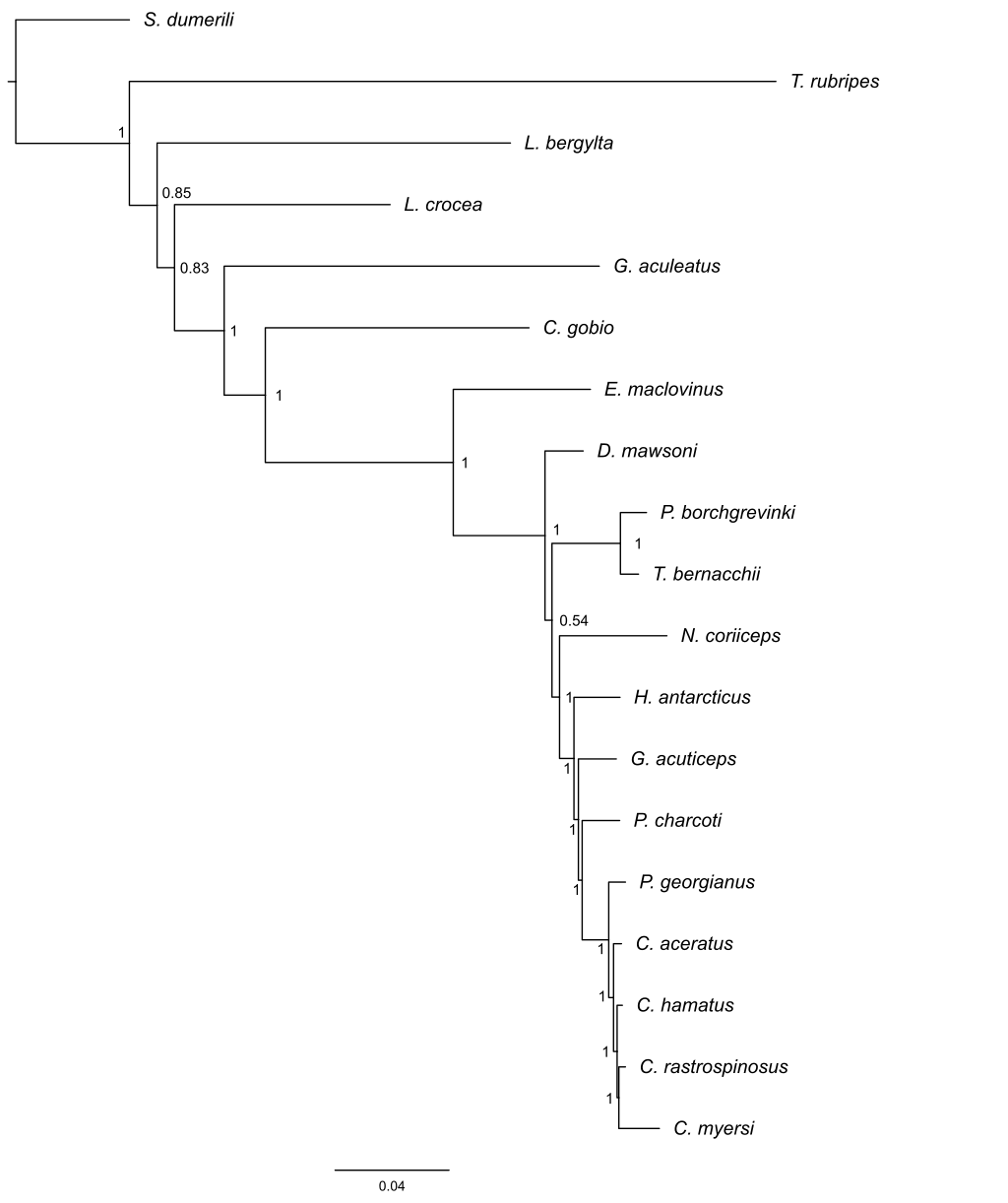


**S Figure 2:**

**Tissue completeness of each notothenioid species’ transcripto**


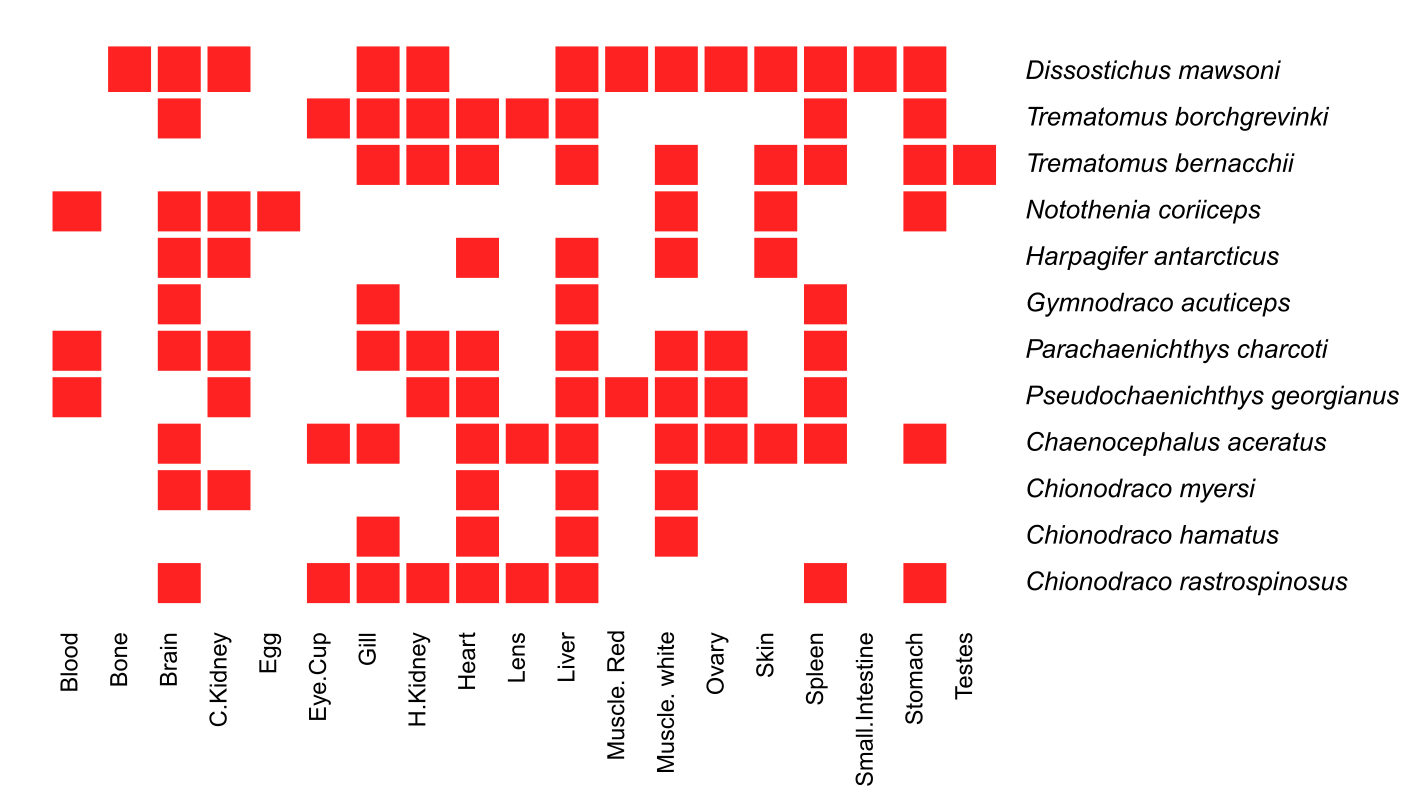


**S Figure 3:**

**Comparison of genomic and transcriptomic derived predicted peptides using BUSCO for three cryonotothenioid species**


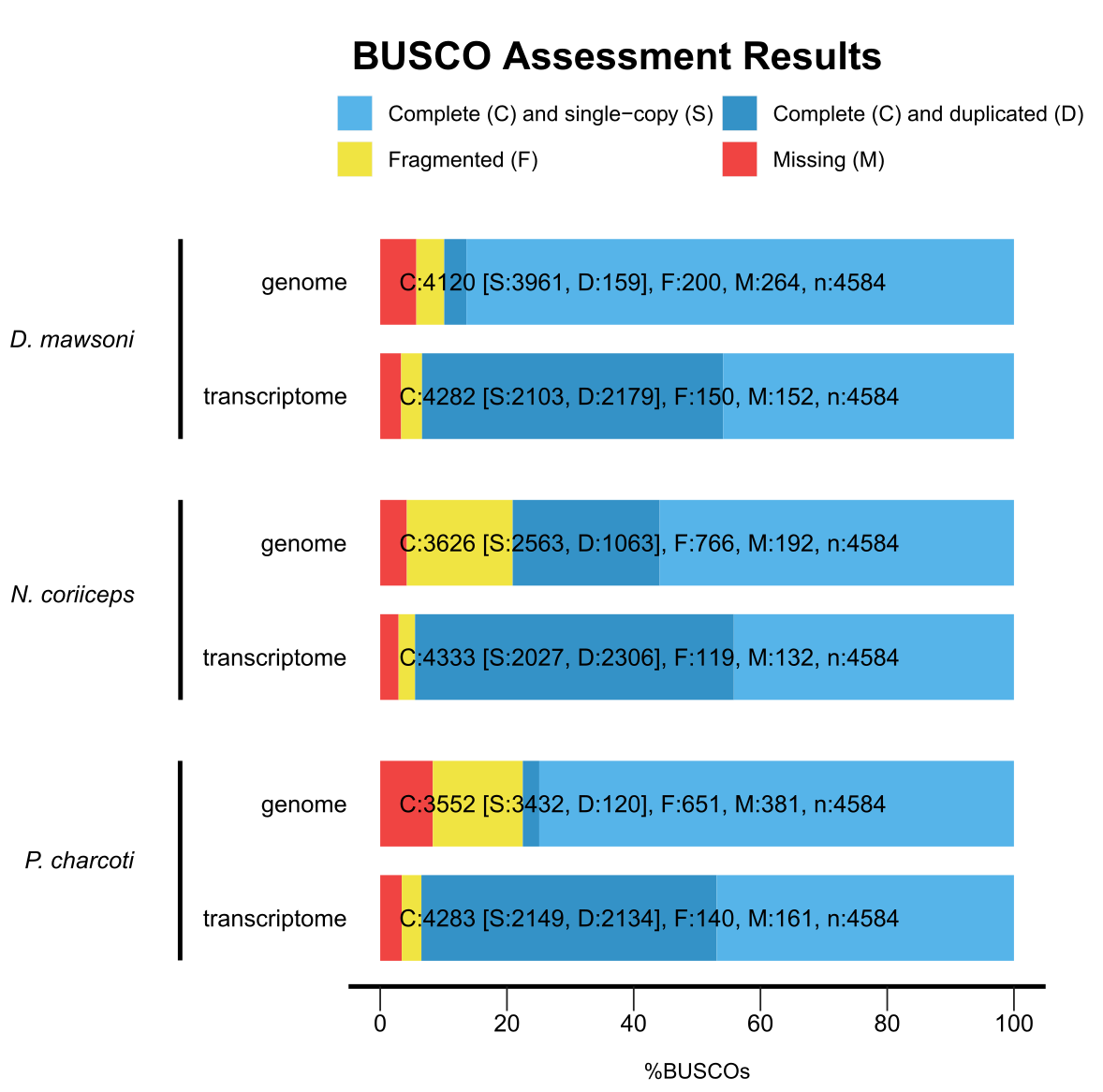

Supplement: evad049_Supplementary_Data [file evad049_supplementary_data.zip › S Figures.docx]
